# Gene body methylation suppresses intragenic transcription and permits epigenetic inheritance in a cnidarian

**DOI:** 10.1038/s41559-026-03090-6

**Published:** 2026-06-02

**Authors:** Lan Xu, Richard Heery, Damir Baranasic, Bojan Žunar, Alvaro Segura Campaña, Vladimir Ovchinnikov, Boris Lenhard, Alex de Mendoza

**Affiliations:** 1 School of Biological and Behavioural Sciences, Queen Mary University of London, London, UK; 2 Centre for Epigenetics, Queen Mary University of London, London, UK; 3 Division of Electronics, Ruđer Bošković Institute, Zagreb, Croatia; 4 MRC Laboratory of Medical Sciences, London, UK; 5 Institute of Clinical Sciences, Faculty of Medicine, Imperial College London, Hammersmith Hospital Campus, London, UK; 6 Laboratory for Biochemistry, Department of Chemistry and Biochemistry, University of Zagreb Faculty of Food Technology and Biotechnology, Zagreb, Croatia; 7 Present Address: Wellcome Sanger Institute, Hinxton, UK

**Keywords:** Evolutionary developmental biology, Epigenetics, Epigenomics

## Abstract

Across invertebrates, DNA methylation is largely restricted to the bodies of highly and constitutively expressed genes. This pattern has led to the widespread hypothesis that gene body methylation regulates gene expression and contributes to environmental adaptation and developmental plasticity, often by analogy to its roles in vertebrate genomes. However, mechanistic evidence testing these ideas remains scarce, limiting the interpretation of epigenetic variation in evolutionary and ecological contexts. Here, using the cnidarian model *Nematostella vectensis*, an early-branching animal which retains an ancestral methylation pattern, we show that loss of DNA methylation produces viable embryos with minimal effects on gene expression. Instead, methylation depletion causes widespread chromatin opening and spurious transcription initiation, particularly from transposable elements embedded within gene bodies. We further demonstrate that methylation is selectively restored in the germline, guided by transcription-associated chromatin, but is not globally reprogrammed after fertilization. As a result, aberrant methylation states can be inherited across generations. These findings identify gene body methylation as an evolutionarily conserved genome defence mechanism, clarify its ancestral function in animal genomes and reveal how incomplete epigenetic resetting can generate heritable regulatory variation with potential evolutionary consequences.

## Abstract (web summary)

Experiments in the cnidarian *Nematostella vectensis* show a role of gene body methylation in transposable element suppression and that epigenetic inheritance is constrained by chromatin context and transcriptional activity.

## Main

A methylated form of the DNA base cytosine, 5-methylcytosine (5mC), plays fundamental regulatory roles in vertebrates, where it contributes to the long-term silencing of regulatory elements and is essential for processes such as transposable element (TE) repression, genomic imprinting, X-chromosome inactivation and tumour suppression^1,2^. Mechanistically, 5mC can interfere with transcription factor binding at promoters and enhancers or recruit methyl-CpG-binding proteins that attract chromatin remodelling complexes to establish repressive chromatin states^3,4^.

**Fig. 1:**
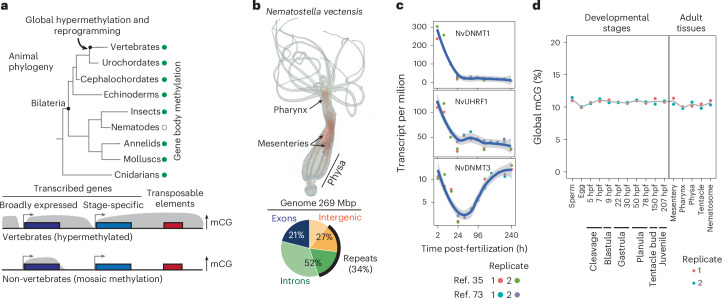
*Nematostella vectensis* exhibits a developmentally stable gbM pattern. **a**, Simplified animal phylogeny showing phyla with gbM (green), those lacking it (white) and the emergence of vertebrate-wide hypermethylation. The lower panel contrasts typical invertebrate and vertebrate methylome features. **b**, Adult anatomy of *Nematostella* alongside key genomic features. Intronic and intergenic regions are subdivided on the basis of the presence (darker green/yellow) or absence of repetitive elements. **c**, Developmental expression profiles of DNMTs and UHRF1 *Nematostella* (Nv) orthologues. Each dot is an RNA-seq replicate from two previous studies^35,73^ and error bands are for a 95% confidence interval. **d**, Global DNA methylation amounts measured with Oxford Nanopore sequencing across developmental stages and adult tissues.

However, vertebrates are exceptional in the animal kingdom: they exhibit global genome hypermethylation, with nearly all CpG sites methylated except at active promoters and enhancers^3,5^. By contrast, most invertebrates show a markedly different methylation landscape, where 5mC is largely confined to the bodies of broadly expressed genes—a distribution incongruent with its canonical role in transcriptional repression^5–7^ (Fig. 1a). The evolutionary transition from a sparsely methylated ancestral state to the hypermethylated vertebrate genome remains poorly understood^8,9^, in part because the functional significance of gene body methylation (gbM) in invertebrates is still unresolved at the molecular level^10–12^. Major invertebrate model organisms such as *Drosophila melanogaster* and *Caenorhabditis elegans* have entirely lost 5mC, leaving a gap in our ability to experimentally interrogate its roles.

Despite this, numerous genomic and ecological studies have linked gbM to key biological processes in invertebrates—including phenotypic plasticity, environmental adaptation and transcriptional stability^13–16^. Yet these associations remain correlative, often assuming that roles described in vertebrates are conserved in invertebrates and comprehensive functional studies are scarce^17–19^. To fully understand the biological relevance of gbM, and how it may have shaped evolution across animals, mechanistic insight is required.

Another major unresolved question relates to how DNA methylation is inherited across generations in animals that apparently lack epigenetic reprogramming during early development. In vertebrates, 5mC patterns are actively re-established during development, most extensively in mammals, but also in lampreys and teleosts^1,2,20,21^. By contrast, invertebrates appear to lack global epigenetic reprogramming events^22^. Although intergenerational inheritance of 5mC has been proposed^23–25^, it remains controversial as disentangling true epigenetic inheritance from genetically encoded methylation patterns is challenging, as DNA sequence variation is a major determinant of methylation in invertebrates^26^. Moreover, it is unclear whether 5mC patterns can be re-established after being perturbed and if there is a functional outcome of aberrant methylation inheritance.

**Extended Data Fig. 1:**
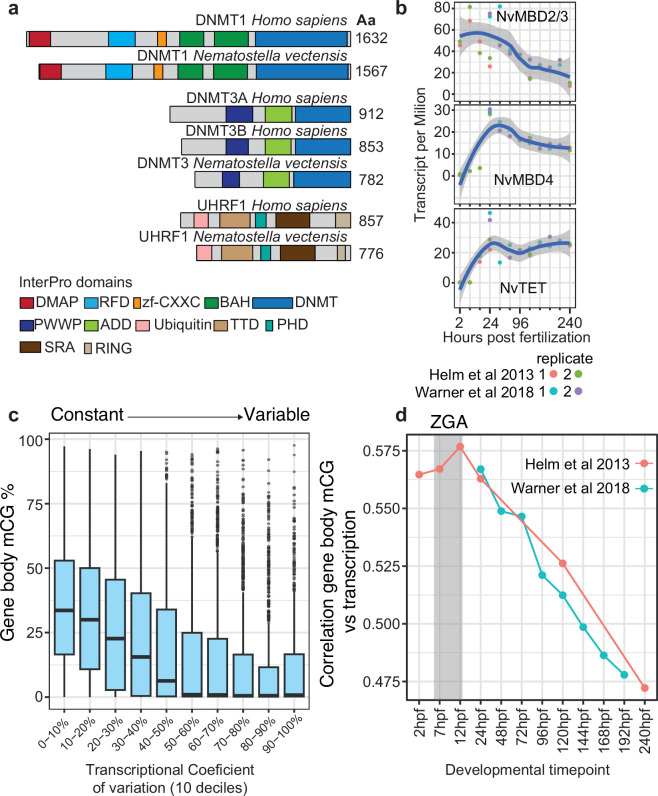
Gene body methylation is associated with transcriptional stability and early developmental expression. **a**) Domain architecture of DNA methylation machinery in *Nematostella* compared to human. Domains are defined according to PFAM/InterPro annotations. Aa: length in amino acids. **b**) Developmental expression dynamics of DNA methylation readers and erasers during *Nematostella* development. Error bands are for a 95% confidence interval. **c**) Distribution of gene body methylation at 25 hpf across genes grouped into deciles based on their transcriptional coefficient of variation throughout development (16,207 genes in total). Boxplots show the median (centerline), interquartile range (box), and the smallest and largest values within 1.5× IQR (whiskers). Values beyond this range are plotted as outliers. **d**) Spearman correlation between gene body methylation levels at 25 hpf and transcript abundance across all genes, using two independent developmental RNA-seq datasets. The grey shaded area marks the timing of zygotic genome activation (ZGA). hpf: hours post-fertilization.

To address these questions, we focused on the cnidarian *Nematostella vectensis*, a tractable model that occupies a key phylogenetic position as an early-branching animal lineage^27^. *Nematostella* exhibits a canonical gbM pattern^22,28^, a relatively simple body plan and features single-copy orthologues of DNA methyltransferase DNMT1, which maintains DNA methylation during replication and DNMT3, which establishes de novo 5mC marks^29^. Both enzymes are structurally conserved with their vertebrate counterparts (Extended Data Fig. 1a). The *Nematostella* genome has evolved slowly^30^, preserves ancestral metazoan linkage groups^31^ and contains a well-characterized repetitive element landscape comprising 34% of the genome^32^ (Fig. 1b). Here we dissect the functional role of gbM in the *Nematostella* genome regulation using a combination of second-generation DNA methylation inhibitors with molecular and epigenomic profiling tools and explore 5mC patterns of inheritance across generations.

## Results

### 5mC depletion is developmentally tolerated in *Nematostella*

In *Nematostella*, the DNA methylation machinery is maternally deposited. Although transcription of the maintenance methyltransferase DNMT1 and its co-factor ubiquitin-like with PHD and RING finger domains 1 (UHRF1) declines rapidly post-fertilization, DNMT3 shows a transient decrease, with expression levels increasing at later developmental stages (Fig. 1c and Extended Data Fig. 1b). However, these transcriptional changes are not reflected in the methylome, with previous studies, across different *Nematostella* strains and profiling techniques reporting stable 5mC amounts throughout development^22,28^. To precisely characterize DNA methylation dynamics in our laboratory population, we densely sampled *Nematostella* developmental stages and adult tissues and profiled global CpG methylation amounts using Oxford Nanopore sequencing with a genome skimming approach^33^. Consistent with earlier findings, global methylation amounts remained largely stable across the life cycle, averaging ~12% (Fig. 1d). This methylation stability observed in *Nematostella* contrasts with the progressive methylation loss observed during development and ageing in bilaterian invertebrates^34^ and may reflect the continuous renewal capacity of cnidarian stem cells and their limited signs of senescence^27^.

We observed in *Nematostella* that genes with high amounts of gbM tend to exhibit the most stable transcriptional profiles across development (Extended Data Fig. 1c), as in other invertebrates^34^. This supports the view that gbM in *Nematostella* is not involved in dynamic gene regulation, but instead marks genes with stable transcription. Moreover, when correlating gbM amounts with gene expression across a developmental RNA-seq time course, the strongest association is observed during zygotic genome activation (7–12 hours post-fertilization (hpf))^35^, after which the correlation progressively weakens (Extended Data Fig. 1d). This temporal decoupling between gbM and transcriptional activity mirrors patterns previously described in annelids^34^, reinforcing the concept that gbM is a relatively static feature linked to early developmental transcription rather than regulatory plasticity.

**Extended Data Fig. 2:**
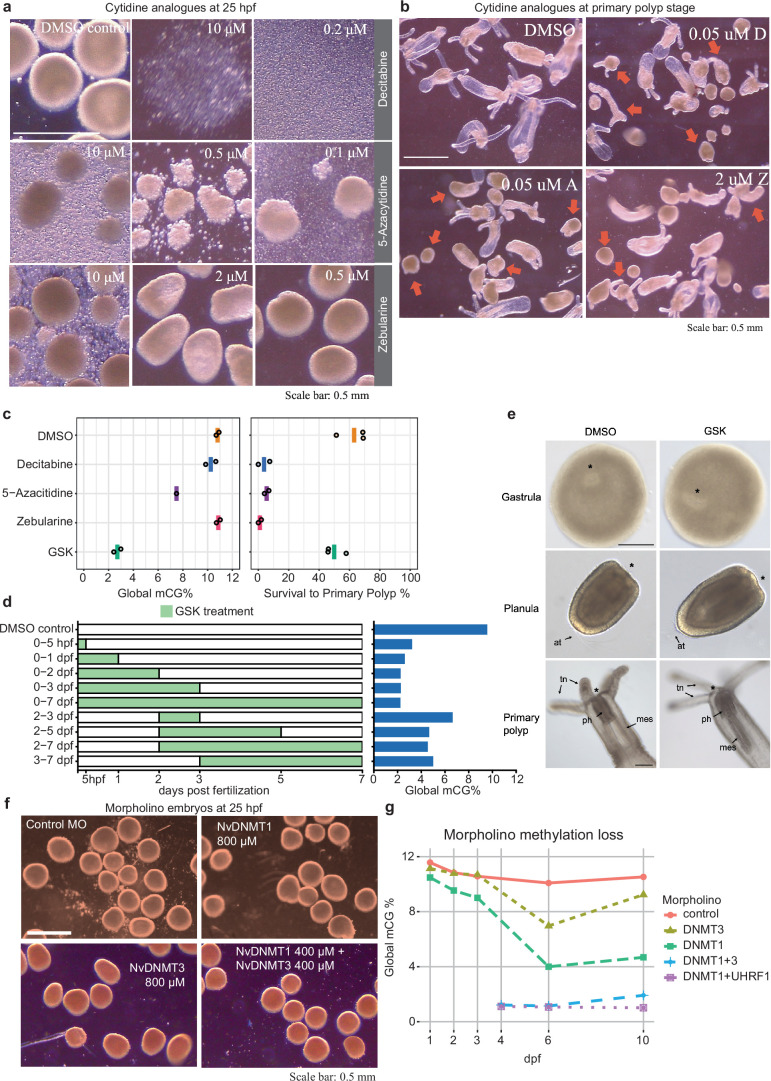
GSK and DNMT-targeting morpholinos reduce DNA methylation with minimal phenotypic effects. **a–b**) Micrographs of *Nematostella* gastrulae (**a**) and primary polyps (**b**) treated with various concentrations of cytidine analogues. Red arrows indicate individuals with arrested or abnormal development. D = Decitabine, A = 5-Azacytidine, Z = Zebularine. **c**) Global DNA methylation levels in gastrula-stage embryos treated with cytidine analogues or GSK, measured by Oxford Nanopore sequencing, alongside corresponding percentage of fertilized eggs that successfully developed into polyps following treatment. Each dot represents a biological replicate and the vertical bars their mean. Concentration used: 0.1% DMSO; 0.05 μM Decitabine; 0.05 μM 5-Azacytidine; 10 μM Zebularine; 5 μM GSK. **d**) Schematic of GSK treatment (5 μM) windows (green boxes) during development, alongside corresponding global methylation levels at primary polyp stage (8 dpf). **e**) Micrographs of gastrula (25 hpf), planula (4 dpf) and primary polyp (8 dpf) treated with DMSO (control) or 5 μM GSK for 25 hours. No morphological defects were observed in the treated group across at least three independent experiments. Asterisks indicate blastopores in gastrula or oral poles in planula and primary polyp. at, apical tuft; tn, tentacle; ph, pharynx; mes, mesentery. Scale bar 100 μm. **f**) Micrographs of gastrulae from eggs injected with translation-blocking morpholinos targeting DNMTs. **g**) Global methylation levels in embryos and polyps injected with different combinations of DNMT-targeting morpholinos. Morpholino concentrations as in panel e.

To investigate the functional role of 5mC, we aimed to deplete DNA methylation using both chemical and genetic approaches. Initial treatments with cytidine analogues—including 5-azacytidine, zebularine and decitabine—resulted in high toxicity, with widespread developmental arrest and poor survival to the juvenile polyp stage even at very low concentrations (Extended Data Fig. 2a–c). Although some depletion of mCG was observed, the effect was modest (Extended Data Fig. 2c). Cytidine analogues are known to induce off-target effects, including DNA/RNA incorporation leading to cytotoxicity, thus confounding the consequences of methylation loss^34,36^. We next turned to GSK-3484862 (hereafter, GSK), a second-generation DNMT1 inhibitor which blocks its catalytic domain and promotes degradation with reduced off-target effects in mammalian cells^37^. Fertilized eggs treated with GSK for as few as 5 h exhibited remarkable global mCG reduction, whereas embryos treated for 25 h exhibited a sixfold reduction in global mCG amounts at as early as gastrula stage (Extended Data Fig. 2d). There were no overt morphological defects and treated embryos reached the primary polyp stage with survival rates comparable to dimethyl sulfoxide (DMSO) controls (Extended Data Fig. 2e), suggesting that depletion of DNA methylation does not interfere with normal development in *Nematostella*.

To compare with genetic perturbation, we injected translation-blocking morpholinos (MOs) targeting DNMT1, DNMT3 and UHRF1. These treatments also led to global methylation loss, although depletion occurred later in development (after 3 days post-fertilization (dpf)) and similarly did not affect survival (Extended Data Fig. 2f,g). These results suggest that maternally deposited DNMT1 messenger RNA/protein is abundant in eggs and directly inhibiting DNMT1 protein with GSK can achieve demethylation earlier than blocking its translation by MO injection.

### Loss of gbM alters chromatin accessibility

**Fig. 2:**
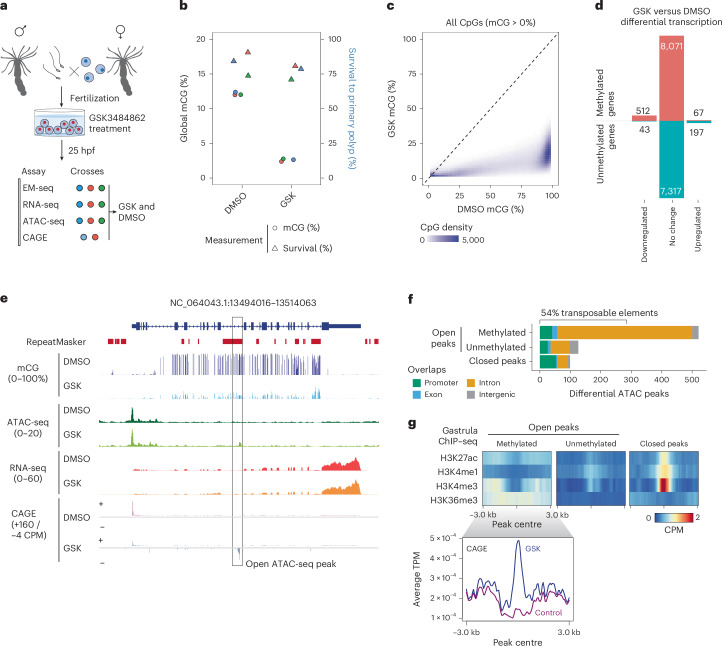
Loss of DNA methylation activates spurious regulatory elements within gene bodies. **a**, Experimental design: fertilized zygotes were treated with GSK (the DNMT1 inhibitor) or DMSO (control) across three independent biological replicates (shown in different colours) and sampled at the gastrula stage for multi-omic profiling. **b**, Global DNA methylation amounts in gastrula samples from all three crosses (shown as circles), quantified using EM-seq, alongside survival percentage to primary polyp stage (triangles). Each dot represents a biological replicate, with colour fillings indicating crosses as in **a**. **c**, Heatmap of 5mC amounts at individual CpGs in control (DMSO) versus GSK-treated samples. Only CpGs with >0% mCG in controls are shown. **d**, Number of differentially expressed and stably expressed genes at the gastrula stage. Genes normally methylated (≥10% mCG in control) are shown in red; normally unmethylated genes (<10% mCG) in blue. **e**, Genome browser snapshot of a representative gene showing ectopic ATAC-seq and CAGE-seq signal in the GSK condition. The RepeatMasker track indicates positions of TEs. The box highlights coordinates of differentially accessible ATAC-seq peaks. **f**, Number of differentially accessible ATAC-seq peaks after GSK treatment, stratified by direction of change and original methylation status. Peaks that close on treatment are primarily located in originally unmethylated (<20% mCG) regions. **g**, Average amounts of histone modifications and CAGE-seq signal at differentially accessible ATAC-seq peaks (as classified in **f**). Histone ChIP–seq data for gastrulae from ref. ^40^, shown as CPM.

**Extended Data Fig. 3:**
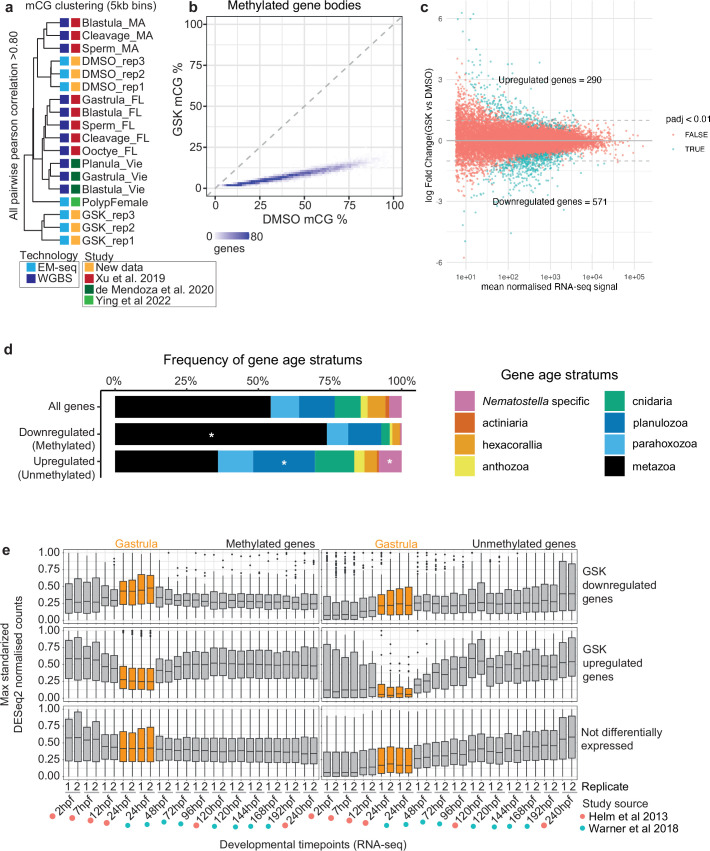
Demethylation induces modest, heterochronic transcriptional changes. **a**) Clustering of genome-wide methylome profiles using 5 kb windows. Methylation data were generated using EM-seq or WGBS, with technique and original study indicated by two color codes in the legend. Distances were computed as 1 – Pearson correlation. Genetic backgrounds: MA = Massachusetts, FL = Florida, Vie = Vienna. In yellow the EM-seq samples generated in this study. **b**) Scatterplot of gene body methylation levels in control versus GSK-treated gastrulae, restricted to genes that are normally methylated (≥10% mCG in control). **c**) MA plot of RNA-seq comparing control and GSK-treated gastrulae (25 hours post-fertilization). Differentially expressed genes (identified by DESeq2 using a Wald test with FDR-adjusted p-values < 0.01) are highlighted in green. Horizontal dashed lines indicate fold changes of 2 and -2. **d**) Stacked bar plots showing the frequency of gene age strata for all genes, downregulated methylated genes, and upregulated unmethylated genes. Asterisks indicate significant enrichment compared to the distribution of all genes (one-sided Fisher’s exact test, raw p-values < 0.05). **e**) Dynamics of standardized expression levels for GSK differentially expressed genes (512 methylated and downregulated, 43 unmethylated and downregulated, 67 methylated and upregulated, 197 unmethylated and upregulated, 8,071 methylated and not differentially expressed and 7,317 unmethylated and not differentially expressed) during normal development. Differentially expressed genes are grouped by methylation status in control conditions. Gastrula stages are highlighted in orange. Data sources are indicated by colored circles, as defined in the legend. Boxplots show the median (centerline), interquartile range (box), and the smallest and largest values within 1.5× IQR (whiskers). Values beyond this range are plotted as outliers.

To investigate the regulatory impact of DNA methylation loss, we focused on the gastrula stage (25 hpf), a timepoint when the correlation between gbM and transcription is still high (Extended Data Fig. 1d), whereas overall cell-type complexity is still lower than in later developmental stages^38^. This stage provides a simplified developmental context to assess transcriptional and epigenomic responses to demethylation. We performed three independent crosses, treating half the fertilized eggs with GSK and the other half with DMSO as control in each replicate(Fig. 2a). We profiled methylation (enzymatic methyl sequencing (EM-seq)), chromatin accessibility (assay for transposase-accessible chromatin using sequencing (ATAC-seq)) and transcription (RNA sequencing (RNA-seq)) from matched samples (Fig. 2a). EM-seq confirmed an approximately sixfold reduction in global 5mC amounts in GSK-treated embryos (from 12% to ~2%), with widespread and uniform depletion across CpGs and gene bodies (Fig. 2b,c and Extended Data Fig. 3b). Methylome clustering, including published datasets^22,28,39^, showed that genetic background remains the primary driver of variation, but GSK-treated samples formed a distinct and highly divergent group, reflecting the impact of methylation loss (Extended Data Fig. 3a).

Differential gene expression analysis identified 264 upregulated and 555 downregulated genes in GSK-treated embryos (false discovery rate (FDR) < 0.01; Fig. 2d and Extended Data Fig. 3c). Stratifying genes by their baseline methylation status revealed a clear pattern: downregulated genes were predominantly methylated under control conditions, whereas upregulated genes were mostly unmethylated (Fig. 2d). This observation mirrors findings from DNMT1 knockdowns in the wasp *Nasonia*^17^, suggesting a conserved role in invertebrates. However, only ~6% of the total number of methylated genes were downregulated in GSK-treated embryos, whereas most (~93%) showed no significant transcriptional change. Downregulated methylated genes were primarily enriched for functions related to amino acid metabolism, whereas upregulated unmethylated genes showed weaker functional enrichment, with a couple of genes associated with chromatin-related processes, including H3K4 methylation and CpG binding (Supplementary Table 1). Consistent with these differences, downregulated genes were enriched for older gene ages, whereas upregulated unmethylated genes were biased towards younger gene age classes (Extended Data Fig. 3d)^9^. Developmental expression patterns in untreated *Nematostella* further revealed that GSK-downregulated genes are normally highly expressed at gastrula, whereas GSK-upregulated genes are typically less expressed at this stage (Extended Data Fig. 3e), consistent with methylation removal causing a modest heterochronic shift rather than widespread transcriptional disruption.

**Extended Data Fig. 4:**
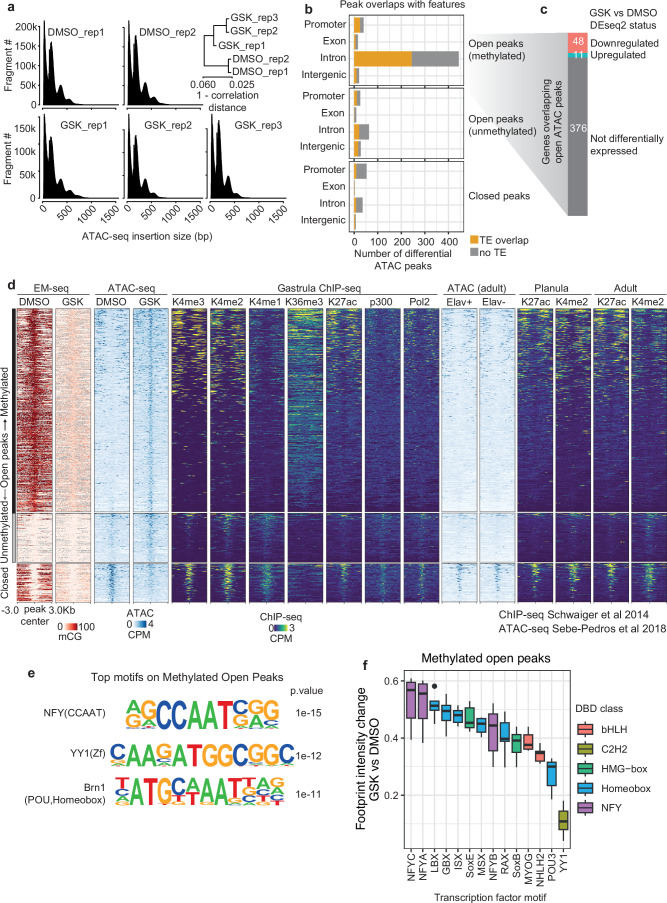
Spurious regulatory activity arises in previously methylated regions upon loss of DNA methylation. **a**) ATAC-seq insertion size profiles for control (DMSO) and GSK-treated gastrulae, showing nucleosome fragment distributions and sample clustering by correlation distance. **b**) Distribution of number of differentially accessible ATAC-seq peaks overlapping with different genomic features. Peaks are categorized based on whether they get opened or closed, and methylation status in control samples (methylated >=20% mCG). Bars indicate whether the regions overlap with Transposable Elements (yellow) or not (gray). **c**) Number of differentially expressed genes between GSK vs DMSO conditions that also contain open ATAC-peaks (originally methylated) in their exons or introns. **d**) Heatmaps showing signal centered on differentially accessible ATAC-seq peaks upon GSK treatment. Shown are methylation (EM-seq), chromatin accessibility (ATAC-seq), and histone modifications or co-activators (ChIP-seq) from gastrulae and later developmental stages. **e**) Top three enriched sequence motifs found by Homer2 in methylated regions that become accessible upon GSK treatment with associated raw p-values. **f**) Transcription factor footprinting analysis at methylated, GSK-induced open peaks. Boxplots show change in footprint intensity (GSK vs DMSO) for selected TF motifs, grouped by DNA-binding domain (DBD) class. Each boxplot contains 6 independent comparisons, computed as DMSO vs GSK per ATAC-seq replicates (for example DMSO replicate 1 vs GSK replicate 1, DMSO replicate 1 vs GSK replicate 2, etc). Boxplots show the median (centerline), interquartile range (box), and the smallest and largest values within 1.5× IQR (whiskers). Values beyond this range are plotted as outliers.

We next assessed chromatin accessibility using ATAC-seq and identified 768 differential peaks (FDR < 0.01), of which 87% became more accessible on GSK treatment (Fig. 2e,f and Extended Data Fig. 4a). We classified these differentially accessible peaks as methylated and unmethylated according to their original methylation amount in controls (≥20% mCG as the threshold). Newly accessible peaks were predominantly located in baseline methylated regions, whereas peaks that lost chromatin accessibility were generally unmethylated and enriched at promoters (Fig. 2f and Extended Data Fig. 4b). Open methylated peaks were largely intronic and rarely affected gene expression: just 13% of genes harbouring new peaks were differentially expressed (48 downregulated and 11 upregulated; Extended Data Fig. 4c). Thus, most chromatin changes due to GSK-mediated demethylation occurred in gene bodies of methylated genes, independent of strong transcriptional changes.

### Intragenic transposons drive spurious transcription on methylation loss

**Extended Data Fig. 5:**
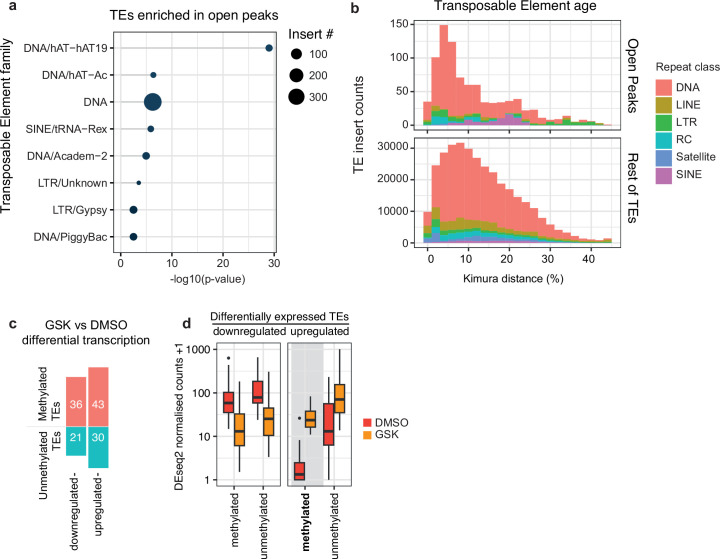
Young DNA transposons contribute to spurious regulatory activity following DNA demethylation. **a**) Enrichment of transposable element families in methylated differentially open chromatin regions (ATAC-seq peaks) upon GSK treatment. Dot size represents the number of insertions, and color indicates statistical significance (two-sided fisher exact test). **b**) Distribution of Kimura distances for TEs overlapping methylated open ATAC-seq peaks (top) versus the full set of annotated TEs (bottom). Colors indicate TE families. **c**) Barplot showing the number of differentially expressed TEs upon GSK treatment, grouped by their methylation status in control conditions and direction of change (upregulated or downregulated). **d**) Boxplots of expression levels for the differentially expressed TEs from panel c in control and GSK-treated conditions, separated by methylation status and expression direction (36 methylated downregulated TEs, 21 unmethylated downregulated TEs, 43 methylated upregulated TEs, and 30 unmethylated upregulated TEs). The grey shaded area highlights distinct derepression of originally silenced and methylated TEs upon demethylation. Read counts are normalised using DEseq2 and TElocal. Boxplots show the median (centerline), interquartile range (box), and the smallest and largest values within 1.5× IQR (whiskers). Values beyond this range are plotted as outliers.

Strikingly, 54% of methylated and open chromatin peaks in GSK treatment group overlapped TEs, particularly DNA transposons and a subset of long terminal repeat retrotransposons (Extended Data Fig. 5a). These TEs were significantly younger than the genome-wide TE population (Extended Data Fig. 5b). Differential expression analysis of individual TE insertions (via TElocal) revealed both upregulated and downregulated elements on GSK treatment (Extended Data Fig. 5c). Only TEs that were originally silenced and methylated showed clear reactivation in an on–off manner on methylation loss. By contrast, changes observed in TEs that were already transcriptionally active were generally quantitative, reflecting shifts in the level of transcription rather than de novo activation (Extended Data Fig. 5d). These results indicate that 5mC plays a selective role in maintaining silencing of intragenic methylated TEs.

To assess whether newly accessible chromatin regions on GSK-mediated demethylation could represent latent or future regulatory elements, we then profiled these regions using existing histone modification datasets^40^. We observed that open methylated peaks were not enriched for canonical enhancer marks in gastrula or later stages, but did overlap with H3K36me3, a gene body-associated mark (Fig. 2g and Extended Data Fig. 4d). By contrast, closing peaks corresponded to normally active promoters, marked by ATAC-seq and H3K4me3 signals (Fig. 2g and Extended Data Fig. 4d). A small number of newly accessible unmethylated regions also overlapped promoter-like regions, but these changes appear unrelated to methylation status.

Motif analysis of open methylated peaks revealed significant enrichment for canonical promoter-binding transcription factors, including NFY, YY1 and Pou domain factors (Extended Data Fig. 4e). Differential footprinting analysis confirmed enhanced NFY occupancy in GSK-treated samples, alongside motifs for developmental regulators (Extended Data Fig. 4f). These findings suggest that demethylation enables these regions to be accessible to transcription factor binding, with some sites exhibiting features typical of promoter architecture.

**Extended Data Fig. 6:**
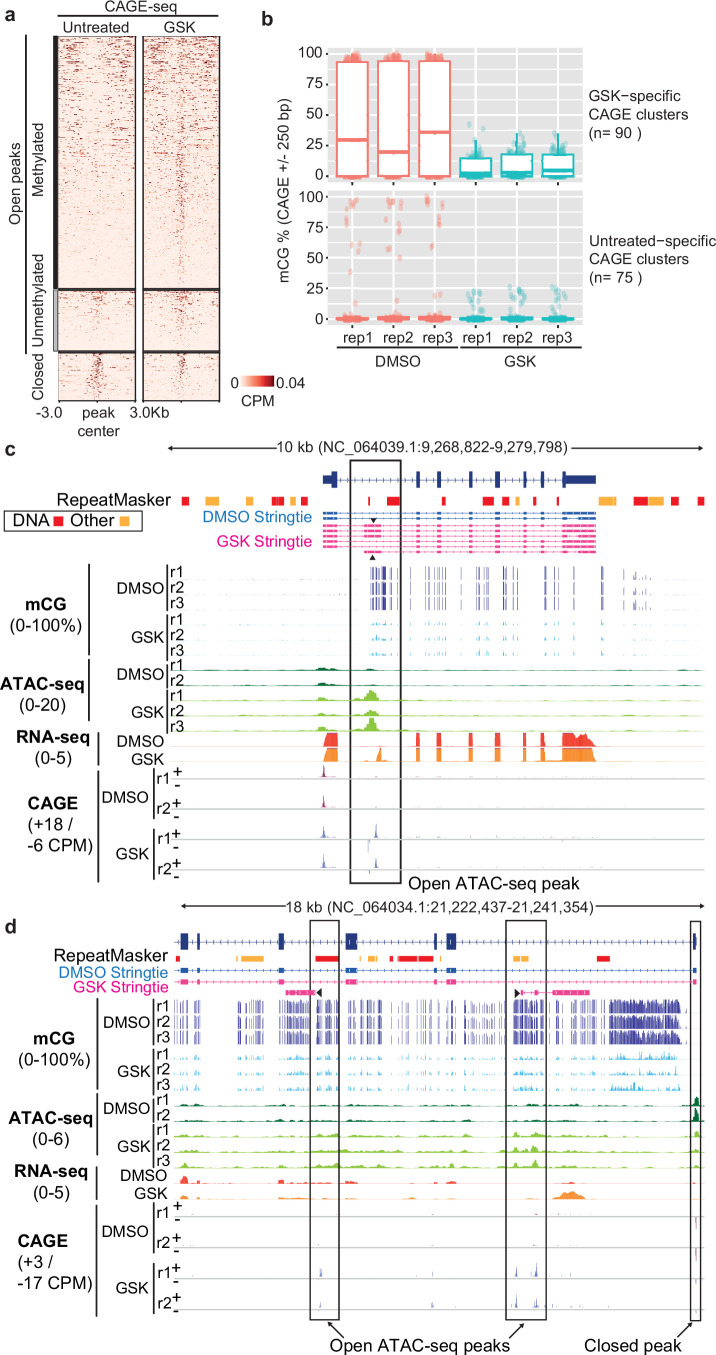
Loss of gene body methylation leads to ectopic transcriptional initiation. **a**) Heatmaps of CAGE-seq signal (Counts Per Million) centered on differentially accessible ATAC-seq peaks in control and GSK-treated gastrulae. Peaks are grouped by their methylation status in the untreated condition and accessibility change in GSK condition. **b**) DNA methylation levels (mCG %) at CAGE clusters specific to GSK-treated (top) or untreated (bottom) embryos, measured in 500 bp windows centered on CAGE signal. Each dot represents an individual cluster in one of three EM-seq replicates. Boxplots show the median (centerline), interquartile range (box), and the smallest and largest values within 1.5× IQR (whiskers). Values beyond this range are plotted as outliers. **c–d**) Genome browser snapshots showing representative examples of GSK-induced transcriptional activation from within previously methylated gene bodies. Tracks include methylation levels (mCG %), chromatin accessibility (CPM), RNA expression (CPM), and CAGE-seq signal (positive CPM for forward strand, negative CPM for reverse strand). RepeatMasker annotations and Stringtie transcript reconstructions are shown above, with DNA transposons marked in red while other repeats in orange. Boxes highlight open and closed chromatin regions with ectopic and reduced CAGE signal, respectively.

To directly test whether methylation loss leads to aberrant transcription initiation, we performed cap analysis of gene expression sequencing (CAGE-seq), which captures the 5′ ends of transcripts^41^ (Fig. 2g). We observed GSK-specific CAGE signal overlapping open methylated peaks, absent in controls (Fig. 2g and Extended Data Fig. 6a). The GSK-specific CAGE transcriptional start site clusters were preferentially located in regions methylated under control conditions, whereas control-specific clusters occurred in unmethylated regions (Extended Data Fig. 6b). Transcriptome assembly revealed that these new transcription start sites (TSSs) often gave rise to shorter isoforms or antisense transcripts. Many of these sites originated from TEs, forming chimaeric transcripts that combine TE-derived sequence with host genes (Extended Data Fig. 6c,d).

Together, these results indicate that gbM prevents spurious regulatory activity and cryptic transcription initiation within actively transcribed genes.

### Partial recovery of DNA methylation occurs in the germline

**Fig. 3:**
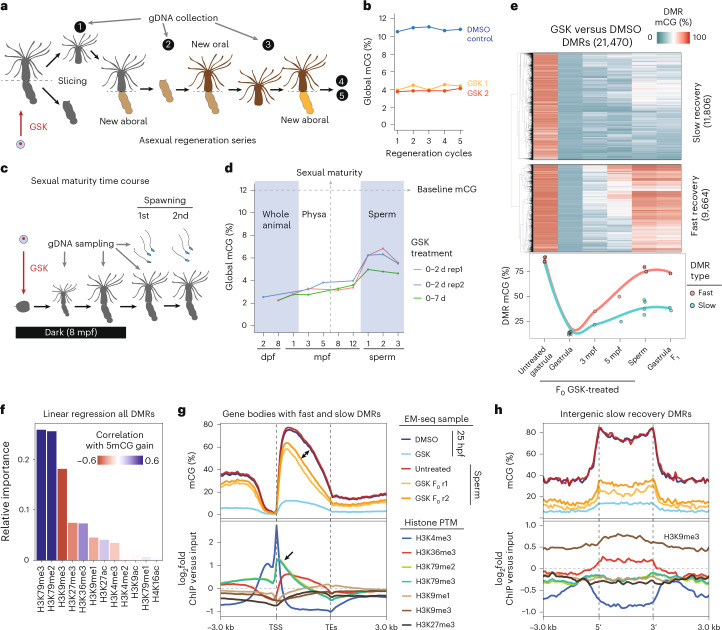
DNA methylation is restored in the germline in transcriptionally active regions. **a**, Schematic of the asexual regeneration experiment performed on demethylated individuals treated with GSK during early development. Animals were bisected and regeneration occurred over ~3 weeks. The newly regenerated half was then repeatedly bisected and regenerated across five successive rounds. **b**, Global DNA methylation amounts measured by Oxford Nanopore sequencing during the regeneration series, shown for two individuals. **c**, Schematic of the growth and spawning timeline of GSK-treated individuals. Genomic DNA was collected at several timepoints and sperm was sampled repeatedly after sexual maturation. **d**, Global methylation amounts during growth and in sperm, assayed via ONT. Each line represents a different treatment batch. Samples at 2 dpf and 8 dpf and 1 month post-fertilization (mpf) were whole animals, whereas physa at 3–12 mpf and sperm were sampled from the same individual over time and spawnings, respectively. **e**, Heatmap and average methylation profiles across development at DMRs identified in GSK versus DMSO gastrulae (Fig. 2c). Fast- and slow-recovery DMRs F_0_ sperm and F_1_ gastrula are clustered using *k*-means clustering. **f**, Regression model showing the contribution of individual histone modifications to methylation recovery at DMRs. Bars are coloured by direction of correlation: blue for positive and red for negative. **g**, Average DNA methylation and histone post-translational modification (PTM) amounts (from ref. ^42^) on gene bodies containing both fast- and slow-recovering DMRs (Extended Data Fig. 8b). Recovery of 5mC in sperm is skewed to the 5’ of the gene body (arrow), where H3K79me2/3 is enriched. EM-seq samples are colour-coded; sperm from individuals treated with GSK in early development is labelled as GSK-F_0_. **h**, Average DNA methylation and histone modification amounts in intergenic regions containing clusters of slow-recovering DMRs.

Although GSK treatment was restricted to early embryogenesis, reduced 5mC amounts persisted into adulthood. To assess whether methylation could be restored over time and whether prolonged loss had functional consequences, we leveraged the regenerative capacity of *Nematostella*^27^. Individual polyps were bisected and the aboral half (physa) was allowed to regenerate a new oral end over ~3 weeks (Fig. 3a). This regenerated animal was then bisected again, this time with a new oral region allowed to regenerate. We repeated this cycle of bisection with alternate aboral/oral regeneration for five successive rounds (Fig. 3a), with DNA collected at each regeneration stage. Despite extensive cell division, regeneration was not impaired compared with controls and global methylation amounts remained low and stable, indicating that somatic mitotic proliferation does not drive methylation recovery or incur detectable fitness costs under stress conditions (Fig. 3b).

**Extended Data Fig. 7:**
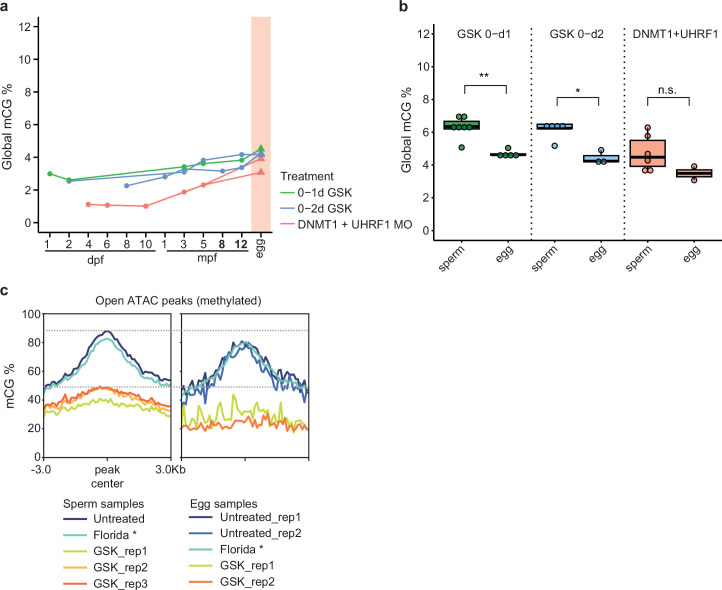
Modest DNA methylation recovery in eggs. **a**) Global DNA methylation levels (measured by Oxford Nanopore sequencing) across developmental timepoints and in egg samples. Each line represents different treatments, either with GSK during early development (1 day in blue, 2 days in green) or with morpholinos targeting DNMT1 and UHRF1. Red shading indicates egg samples from sexually mature adults. **b**) Comparison of global DNA methylation levels in sperm and eggs of individuals from the same group derived from early GSK-treated or morpholino-injected embryos. Each dot represents gametes from one individual. Asterisks indicate statistical significance for a two-sided Wilcoxon rank sum test with *, ** and *** indicating FDR-adjusted p-values less than 0.05, 0.01 and 0.001, respectively and n.s. p-values over 0.05. Boxplots show the median (centerline), interquartile range (box), and the smallest and largest values within 1.5× IQR (whiskers). **c**) Average methylation level profiles on methylated open ATAC-seq peaks in sperm (left) and eggs (right) of control and GSK-treated individuals.

Similarly, animals sampled throughout development and allowed to reach sexual maturity exhibited evidence of minimal methylation recovery (Fig. 3c,d). By contrast, sperm samples obtained from successive spawnings showed a substantial increase in global 5mC, reaching ~50% of baseline levels (~6% versus 12%; Fig. 3d), whereas eggs showed only modest recovery (Extended Data Fig. 7a,b). These results indicate that de novo methylation is responsible for restoring 5mC and that this recovery is more pronounced in the male germline, consistent with its slightly higher baseline methylation amounts even under control conditions (Fig. 1d and Extended Data Fig. 7b,c).

To investigate the genomic distribution of this partial methylation recovery, we defined differentially methylated regions (DMRs) between control and GSK-treated gastrulae as the baseline coordinates of methylation loss. We then tracked methylation amounts at these DMRs across development and in sperm (Fig. 3e). Unsupervised clustering of DMRs revealed two major classes: one set exhibited slow or no recovery, whereas the other regained near-control amounts of methylation in sperm (Fig. 3e). These results indicate that de novo methylation is not uniformly targeted across the genome, but instead exhibits region-specific preferences.

### Transcription-coupled chromatin features predict methylation recovery

**Extended Data Fig. 8:**
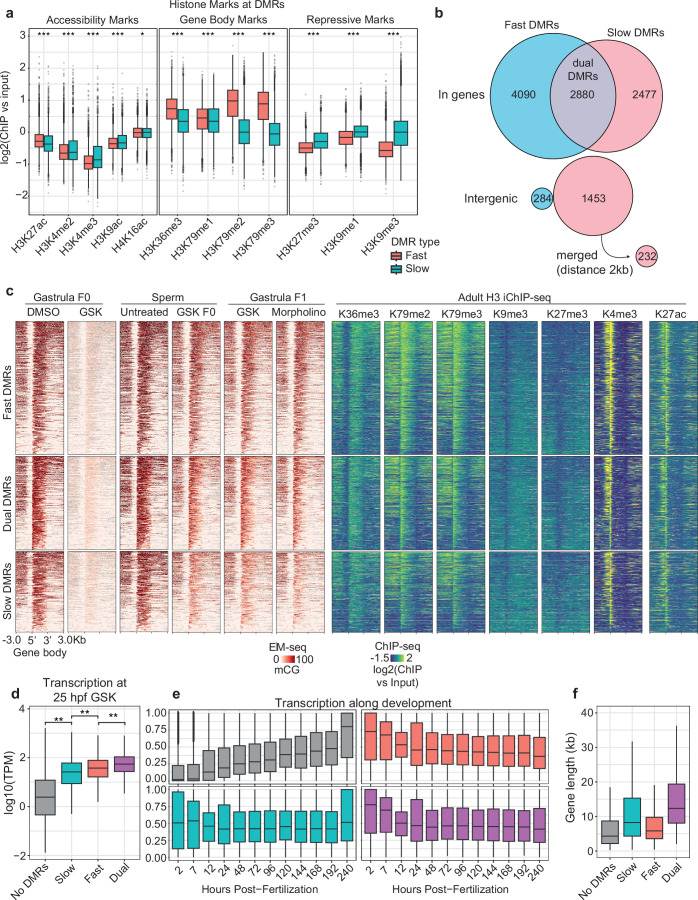
Chromatin context and expression dynamics influence the rate of DNA methylation recovery. **a**) Boxplots showing the distribution of histone ChIP-seq signal at fast and slow-recovering DMRs. Histone modifications are grouped into three functional categories: accessibility marks (left), gene body-associated histone modifications (middle), and repressive marks (right). Asterisks indicate statistical significance with a two-sided Wilcoxon rank sum test with *, ** and *** indicating FDR-adjusted p-values less than 0.05, 0.01 and 0.001, respectively. Adult Nematostella ChIP-seq from^42^. Boxplots show the median (centerline), interquartile range (box), and the smallest and largest values within 1.5× IQR (whiskers). Values beyond this range are plotted as outliers. **b**) Venn diagram showing the number of genes overlapping fast (blue) or slow (red) DMRs, as well as DMRs located in intergenic regions. Intergenic slow DMRs were further clustered by merging regions within 2 kb of each other. In 85% of dual DMR genes, the fast DMR is 5’ of the slow DMR in the same gene body. **c**) Heatmaps of DNA methylation (EM-seq) and histone ChIP-seq signal across gene bodies classified as overlapping fast, slow or dual (fast & slow) DMRs. Shown are methylation levels in gastrula (F0), sperm, and F1 gastrula from GSK-treated and DNMT1 + UHRF1 morpholino-injected progenitors. **d**) Transcription levels (log10 TPM) for genes containing no DMRs, fast, slow, or dual DMRs in GSK-treated gastrulae at 25 hpf (9,784 No DMR genes with numbers of other categories given in panel b). Asterisks indicate raw p-values less than 0.01 with a two-sided Wilcoxon rank sum test. Boxplots show the median (centerline), interquartile range (box), and the smallest and largest values within 1.5× IQR (whiskers).**e**) Standardised transcription dynamics of genes with fast (red), slow (blue), dual (purple), or no (grey) DMRs (numbers of DMRs as in panel d) across development. RNA-seq data from two previous studies,^70,74^. Boxplots show the median (centerline), interquartile range (box), and the smallest and largest values within 1.5× IQR (whiskers). **f**) Distribution of gene length for genes overlapping no DMRs, fast, slow, or dual DMRs (numbers of DMRs as in panel d). Boxplots show the median (centerline), interquartile range (box), and the smallest and largest values within 1.5× IQR (whiskers).

To understand what determines differential methylation recovery across the genome, we examined the chromatin context of fast- and slow-recovering DMRs^42^. DMRs that regained methylation rapidly were enriched for gene body-associated histone marks, including H3K79me2/3 and H3K36me3 (Extended Data Fig. 8a). By contrast, slow-recovering DMRs were enriched for repressive modifications such as H3K9me3 and H3K27me3 (Extended Data Fig. 8a). To test whether histone features alone could predict methylation recovery, we trained a linear regression model using the genome-wide histone chromatin immunoprecipitation sequencing (ChIP–seq) data. The model explained a substantial proportion of the variance in recovery (*R*^2^ = 0.53), with gene body marks positively associated with remethylation and repressive marks showing strong negative associations (Fig. 3f). Notably, H3K79me2/3 emerged as a stronger predictor of recovery than H3K36me3, despite the known interaction of the PWWP domain of DNMT3 with H3K36me3^43^, suggesting an underappreciated role for H3K79 methylation in recruiting de novo DNA methylation in animals.

Given the association between gene body histone marks and transcription, we next investigated whether gene expression levels correlate with methylation recovery. We classified genes by the type of DMRs they contained: fast, slow or both (dual) (Extended Data Fig. 8b). All three gene categories were marked by active chromatin features such as gene body H3K36me3 and promoter H3K4me3, although genes in the slow DMR category also showed moderate enrichment for repressive marks (Extended Data Fig. 8c). Genes with fast or dual DMRs showed significantly higher transcriptional levels in the gastrula stage than genes with only slow DMRs (Extended Data Fig. 8d) and were generally more highly expressed during early development under control conditions (Extended Data Fig. 8e). This suggests that transcriptionally active genes, especially those involved in early developmental programmes, are preferentially targeted for methylation restoration.

Interestingly, genes containing both fast- and slow-recovering DMRs were significantly longer than those with only fast-recovering regions (Extended Data Fig. 8f). This allowed us to explore spatial differences in methylation recovery along their gene bodies. In these dual DMR-harbouring genes, methylation recovery was faster at the 5′ end, coinciding with the peak enrichment of H3K79me2/3, whereas methylation recovered more slowly towards the 3′ end (Fig. 3g and Extended Data Fig. 8c). This 5′-biased recovery did not align with the more uniform distribution of H3K36me3 along gene bodies, suggesting that H3K36me3 is not the sole instructive mark guiding DNMT3 activity. Instead, the spatial pattern implies that methylation recovery proceeds quantitatively during transcriptional elongation: highly transcribed shorter genes are more rapidly remethylated, whereas longer genes require more time to fully re-establish methylation across their entire length.

To assess recovery dynamics in transcriptionally silent regions, we focused on slow-recovering intergenic DMRs. Although relatively fewer in number (1,453), these DMRs were clustered in repeat-rich, gene-poor regions of the genome and were strongly enriched for H3K9me3 while being depleted of active histone marks such as H3K4me3 (Fig. 3h). These heterochromatic features suggest a chromatin environment that resists remethylation, suggesting that methylation in these regions is normally maintained by DNMT1, rather than actively re-established by transcriptionally dependent DNMT3. Together, these findings indicate that methylation recovery in *Nematostella* is tightly coupled to transcription and chromatin context. Highly transcribed gene bodies regain methylation efficiently, whereas repressive or heterochromatic regions remain demethylated, revealing a selective and context-dependent mechanism of epigenetic memory following experimentally induced methylation loss.

### Lack of epigenetic reprogramming enables inheritance of aberrant methylation

**Fig. 4:**
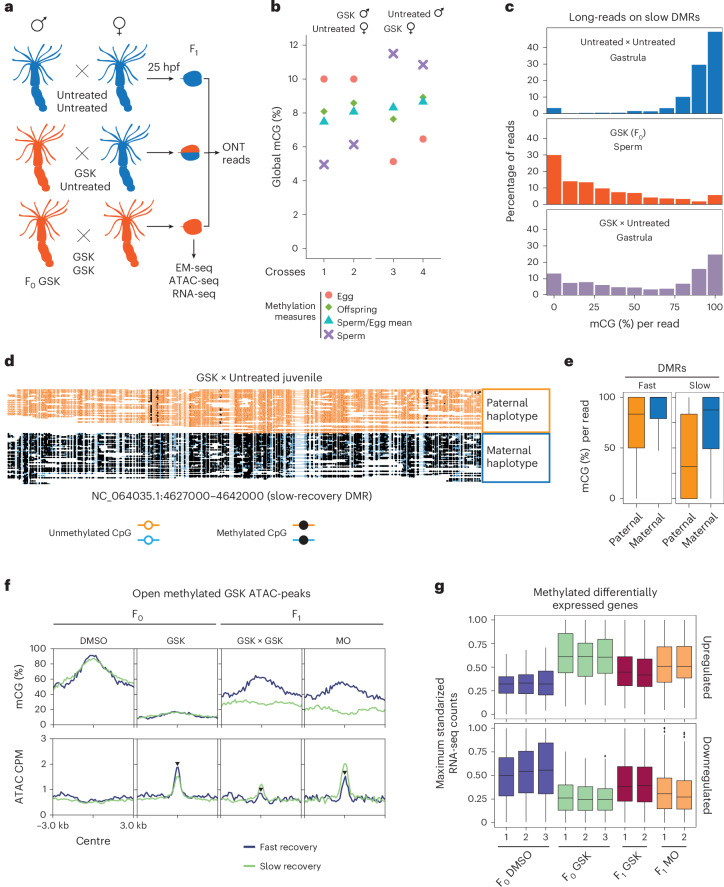
Aberrant DNA methylation patterns are passively inherited in the next generation. **a**, Schematic of genetic crosses between GSK-treated F_0_ and untreated F_0_ individuals. Embryos of F_1_ at the gastrula stage were collected for multi-omic profiling. **b**, Global DNA methylation amounts measured by Oxford Nanopore sequencing in four independent crosses. GSK-treated males with the lowest methylation amounts were selected for crossing. Eggs (red circle), sperm (purple cross) and resulting offspring (green diamond) are shown. The mean methylation of the parental gametes is represented by a blue triangle. **c**, Distribution of per-read methylation amounts at slow-recovery DMRs across two different crosses. Control gastrulae are shown in blue, GSK-F_0_ sperm in orange and F_1_ gastrulae resulting from GSK-F_0_ sperm crossed with control eggs in purple. **d**, Single-molecule Oxford Nanopore methylation at a representative slow-recovery DMR, phased by parental haplotype. DNA was obtained from a juvenile derived from a GSK-F_0_ sperm × untreated egg cross. Unmethylated CpGs of paternal origin are shown in orange, maternal in blue and methylated CpGs as black circles. **e**, Per-read methylation distribution for fast- and slow-recovery DMRs (9,664 and 11,806 DMRs), separated by parental origin in the same individual as in **d**. Boxplots show the median (centre line), interquartile range (IQR) (box) and the smallest and largest values within 1.5× IQR (whiskers). **f**, Average ATAC-seq signal (as CPM) and DNA methylation over ectopic open peaks (defined in Fig. 2f) that overlap fast- or slow-recovery DMRs. Gastrula-stage F_1_ embryos were derived from either GSK-F_0_ crosses or DNMT1 + UHRF1 MO-injected F_0_ crosses. **g**, Standardized transcription amounts of methylated, differentially expressed genes (as defined in Fig. 2d; 512 downregulated and 67 upregulated) in F_1_ gastrulae derived from GSK-treated or MO-injected F_0_ progenitors. Each number above group names represents a biological replicate. Boxplots show the median (centre line), IQR (box) and the smallest and largest values within 1.5× IQR (whiskers); values beyond this range are plotted as outliers.

**Extended Data Fig. 9:**
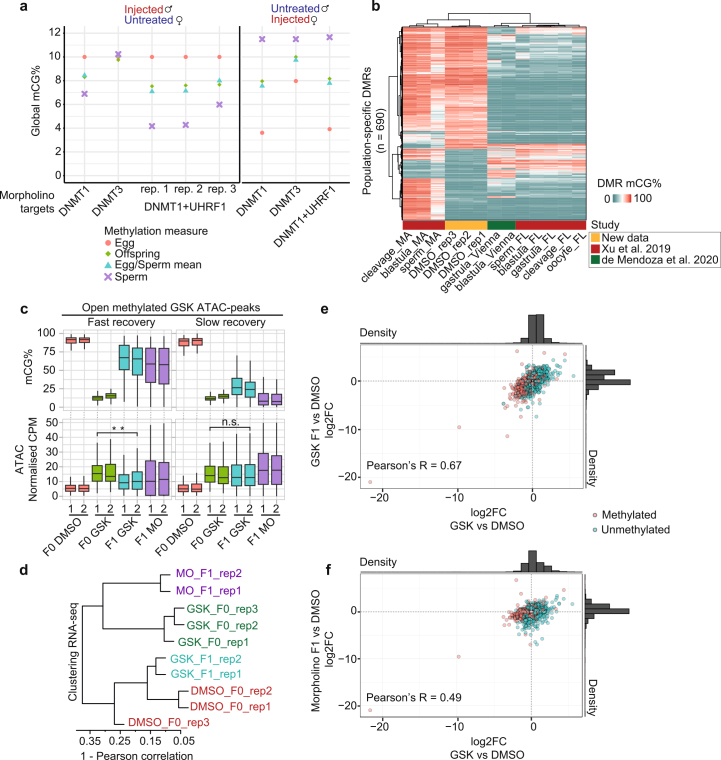
Inheritance and regulatory impact of DNA methylation in the F1 generation. **a**) Global DNA methylation levels in crosses between gametes derived from morpholino-injected parents and untreated partners. Plots show methylation levels in eggs (red circle), sperm (purple cross), offspring (green triangle), and the mean of egg and sperm (blue diamond) for each cross type and replicate. **b**) Heatmap showing the methylation levels on DMRs identified across different Nematostella populations, gathered from previous studies. Massachusetts (MA) and Florida (FL) WGBS samples were from the same study. DMSO represents F0 DMSO gastrulae in this study. Clustering of DMRs and samples using Ward’s clustering method. **c**) DNA methylation and chromatin accessibility (DEseq2 normalised ATAC-seq signal) at GSK-induced methylated open chromatin peaks, stratified by DMR recovery speed (154 fast regions vs. 325 slow regions) and cross type. Bars show data from control or GSK-treated F0 gastrulae and F1 embryos from GSK- or DNMT1 + UHRF1 morpholino-treated parents. Asterisks indicate raw p-values < 0.01 in a two-sided t-test and n.s. p-values over 0.05. Boxplots show the median (centerline), interquartile range (box), and the smallest and largest values within 1.5× IQR (whiskers). **d**) Hierarchical clustering of RNA-seq samples based on Pearson correlation of normalized gene expression profiles using Ward’s clustering criterion. **e–f**) Scatter plots showing log2 fold changes in gene expression between GSK F0 and DMSO conditions versus expression changes in GSK-treated (e) or morpholino-injected (f) F1 embryos against DMSO F0. Genes are color-coded according to their original methylation level in DMSO samples (>10 % mCG as methylated).

To determine whether demethylation-induced epimutations are transmitted to the next generation, we performed reciprocal crosses between selected GSK-treated and untreated animals (F_0_), generating embryos from all combinations of methylated and demethylated gametes (Fig. 4a). All fertilizations were viable. We sampled the resulting offspring (F_1_) and profiled their methylomes using long-read Oxford Nanopore sequencing. Remarkably, global methylation amounts in these offspring consistently approximated the arithmetic mean of the parental gametes, regardless of whether the demethylated contribution came from egg or sperm (Fig. 4b and Extended Data Fig. 9a). This pattern suggests passive inheritance of parental methylation states, without preferential reprogramming or erasure from either parental genome.

To explore this further, we focused on a cross between a demethylated male (with the lowest methylation in the group) and an untreated female (cross 1 in Fig. 4b). We analysed per-read methylation at slow-recovering DMRs, reasoning that these regions would retain the strongest signature of paternal methylation loss. Each read corresponds to a single DNA molecule, thereby capturing the diversity of haplotypes and cell states within the pooled embryo sample. In control embryos, nearly all reads across these loci were heavily methylated (>90% mCG), whereas in sperm of this demethylated male, most reads showed low methylation amounts (<40%; Fig. 4c). By contrast, the cross involving demethylated sperm and control eggs resulted in gastrula-stage embryos with a bimodal methylation distribution (Fig. 4c). This pattern is consistent with maternal-derived reads remaining methylated, whereas paternal-derived reads retaining their hypomethylated state. The co-existence of these epigenetically distinct haplotypes indicates a lack of post-fertilization methylation reprogramming, where a parental methylation pattern would serve as template for the other.

To test whether these differences persist throughout development, we raised F_1_ polyps from this cross and performed haplotype-resolved methylation profiling using homozygous paternal single nucleotide polymorphisms to discriminate reads overlapping heterozygous positions. Even in fully developed juveniles, slow-recovering DMRs on the paternal haplotype remained hypomethylated, whereas the maternal homologue maintained baseline methylation (Fig. 4d,e). By contrast, fast-recovering DMRs regained methylation on both haplotypes (Fig. 4e). These findings confirm that, in *Nematostella*, there is no global erasure or re-establishment of DNA methylation following fertilization. Instead, partial remethylation occurs gradually and selectively, leading to inheritance of epimutations.

To assess whether naturally occurring methylation differences in *Nematostella* reflect similar variability to the experimentally induced epimutations, we identified DMRs between our control samples and three previously published datasets representing distinct colonies and genetic backgrounds. We detected 690 DMRs across these populations, with variation strongly associated with colony of origin rather than developmental stage (Extended Data Fig. 9b). Of the 383 population variable DMRs that overlap the recovery DMRs, 96% overlapped with the slow-recovering DMRs. This suggests that slower recovery, and probably less efficient targeting of DNA methylation, contributes to stable, heritable epigenetic variation among populations.

### Inherited epimutations impact chromatin and transcription in the next generation

Having established that aberrant methylation states are inherited, we next asked whether these epimutations have genome regulatory consequences. We generated F_1_ embryos from GSK-treated or MO-injected (DNMT1/UHRF1-targeting) F_0_ animals and collected gastrula-stage samples for EM-seq, ATAC-seq and RNA-seq (Fig. 4a). We focused on the ‘open methylated peaks’ that had emerged in F_0_ GSK-treated gastrulae (Fig. 2f), ectopic accessible regions arising from previously methylated loci. We classified these peaks on the basis of their overlap with fast- or slow-recovering DMRs and assessed both DNA methylation and chromatin accessibility (Fig. 4f). EM-seq confirmed that peaks overlapping fast DMRs had largely regained methylation in the F_1_, almost to normal amounts, consistently showing reduction of ATAC-seq signal on those regions (Fig. 4f and Extended Data Fig. 9c). By contrast, peaks in slow-recovering DMRs remained hypomethylated and chromatin remained accessible on those regions (Fig. 4f and Extended Data Fig. 9c). This effect was more pronounced in MO-derived F_1_ embryos, which inherited lower global methylation amounts from the previous generation (4.3% mCG versus 5.7–6.1% mCG in F_1_ GSK). These results indicate that persistent hypomethylation alone is sufficient to sustain ectopic chromatin accessibility across generations. In the F_0_ generation, chromatin dysregulation could have been attributed to DNMT1 protein degradation, potentially affecting non-catalytic roles such as recruitment of repressive complexes. However, DNMT1 amounts are not perturbed in F_1_ embryos, demonstrating that chromatin dysregulation occurs independently of DNMT1 loss-of-function effects.

We then examined the transcriptional consequences of inherited methylation states by tracking genes originally dysregulated in F_0_ GSK-treated gastrulae (Fig. 2e). In F_1_s, expression of these genes was intermediate between control and F_0_ demethylated samples (Fig. 4g). MO F_1_s retained more of the dysregulated transcriptional profile, consistent with their lower remethylation. Genome-wide, we observed high concordance between F_1_ and F_0_ transcriptional responses, with MO-derived F_1_s clustering more closely to GSK-treated F_0_s than to controls (Extended Data Fig. 9d–f). This demonstrates that inherited epimutations can shape both chromatin and transcriptional landscapes, raising the possibility that these non-genetic changes could be subject to natural selection.

## Discussion

**Fig. 5:**
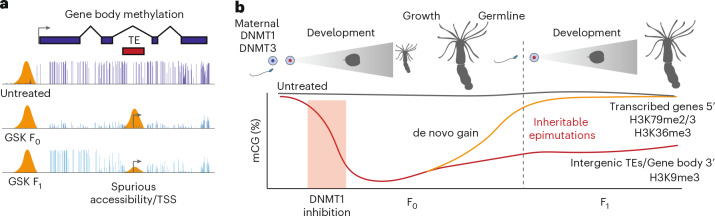
gbM suppresses spurious TSSs and is heritable. **a**, Loss of gbM results in increased chromatin accessibility and the emergence of ectopic TSSs, often overlapping TEs. Recovery of methylation in these regions can suppress these effects. **b**, Model of DNA methylation inheritance in *Nematostella*. Under normal conditions, methylation remains stable (grey line). Following forced demethylation, methylation is rapidly restored in the germline in actively transcribed regions (orange line) but recovers more slowly in the soma and in intergenic regions and at the 3′ ends of gene bodies (red line). This asymmetry in recovery leads to heritable epimutations in the next generation.

We uncover a primary mechanistic role for gbM in invertebrates: the suppression of spurious transcriptional initiation within highly and broadly expressed genes (Fig. 5a). The insertion of TEs into these gene bodies poses a particular threat, as many TEs harbour intrinsic regulatory activity capable of driving ectopic transcription. A silencing mechanism that preserves transcriptional integrity in such regions is therefore important. Unlike heterochromatic marks, such as H3K9me3 that can hinder transcription^44^, DNA methylation appears to offer a more permissive form of repression compatible with active transcription. This functional specialization may reflect a division of labour in animal chromatin, in which the ancestral co-occurrence of heterochromatin and 5mC diversified into separate silencing strategies. In animals, the evolution of targeting domains such as PWWP and ADD in DNMT3 probably allowed de novo methylation to be directed towards transcriptionally active gene bodies^36^. Plants exhibit similar gbM patterns via non-homologous methyltransferases, probably the result of convergent evolution^10,45^. Yet, recent evidence suggests that gbM in plants also serves to suppress intragenic antisense transcription, pointing to a shared functional logic across kingdoms^46^.

Whereas small-RNA pathways such as the PIWI-piRNA system provide targeted, sequence-specific surveillance against TE transcripts, gbM appears to serve a complementary yet distinct function. The piRNA system primarily acts in *trans* to degrade specific TE transcripts and guide localized transcriptional silencing. For example, in *Nematostella* and other cnidarians, piRNAs are enriched during early development and in adult stem cells/germline^47,48^. By contrast, gbM acts in *cis* as a chromatin feature that broadly suppresses spurious transcriptional initiation within actively transcribed regions, including intragenic TEs. Thus, whereas small-RNA pathways primarily combat active transposon mobilization, gbM appears to safeguard the transcriptional integrity of host genes that harbour embedded repetitive elements. Notably, the coupling between small-RNA pathways and DNA methylation deposition is thought to represent a later evolutionary innovation, most clearly established in tetrapods^49^, suggesting that gbM-mediated repression and piRNA surveillance may operate largely independently in invertebrates.

Notably, the relatively modest transcriptional changes observed on gbM loss in *Nematostella* imply that gbM does not play a central role in the dynamic regulation of gene expression. This aligns with previous studies in invertebrates and plants showing weak correlations between gbM and transcriptional changes^11,12,50^, where genetics has more weight explaining methylation variation than transcription^25,26^. Instead, our data support a model in which gbM contributes primarily to transcriptional integrity rather than regulating host gene expression per se. This role mirrors findings in vertebrates, where loss of DNA methylation also triggers spurious intragenic transcriptional initiation^51,52^. However, in vertebrates, global loss of 5mC is typically lethal because of its broader regulatory functions^53^, including promoter and enhancer control as well as extensive TE silencing^1,2^. These expanded roles probably evolved as a consequence of genome hypermethylation, which subsequently necessitated more robust regulatory control and epigenetic reprogramming mechanisms across generations. By contrast, we show that in *Nematostella*, spurious transcripts arising from demethylated gene bodies do not appear to cause overt developmental defects. Viable adults and fertile offspring can still be produced, suggesting that these epimutations can be tolerated. However, in other invertebrates, such as insects, spurious transcription may be more deleterious—for example, by producing dominant-negative isoforms or interfering with transcript stability—potentially explaining why DNMT1 knockdowns are lethal in some species^17,18^, but not so in others^19^.

Although widespread methylation loss was achieved using both inhibitors and MO, residual 5mC remained detectable. Complete elimination of DNA methylation would probably require genetic disruption of both DNMT1 and DNMT3. However, loss of DNMT proteins could introduce additional phenotypes unrelated to methylation, given their known non-catalytic roles. Future genetic approaches targeting catalytic activity specifically will help further dissect the functional consequences of total DNA methylation loss.

We also show that whereas *Nematostella* lacks global post-fertilization epigenetic reprogramming, the germline can partially restore methylation at actively transcribed loci (Fig. 5b). The fact that recovery occurs in the germline but not during somatic mitosis further supports the idea that the primary role of gbM is transposon control, as preserving genome integrity is especially critical in the germline, where most silencing mechanisms (for example, piRNAs) are concentrated. The observed de novo methylation, probably mediated by DNMT3, is preferentially targeted to gene bodies marked by transcription-associated histone modifications, but slow at heterochromatin regions (Fig. 5b). Notably, DNMT3 is expressed both early and late in development (Fig. 1c), yet later developmental expression does not appear sufficient to drive substantial 5mC gain in somatic tissues, even after complete regeneration cycles. This may reflect developmental regulation of DNMT3 activity, for example through restricted nuclear localization or reduced enzymatic activity in somatic cells. Possibly, restricted de novo methylation in somatic tissues may have limited evolutionary consequences, as TE mobilization in the soma is unlikely to be inherited, whereas maintenance by DNMT1 may be sufficient to preserve 5mC at essential, highly expressed genes. After fertilization, however, no widespread reprogramming occurs: the distinct epigenetic states of parental genomes persist well into embryogenesis and even adulthood, allowing for the inheritance of epimutations across generations. Over time, inherited hypomethylation in gene bodies may be corrected through transcription-dependent remethylation, gradually restoring epigenetic stability. However, persistent epimutations can have regulatory consequences, including ectopic chromatin accessibility and the emergence of new transcripts. Whereas most of these are probably neutral or deleterious, a subset could reveal previously silenced regulatory or coding potential, creating opportunities for evolutionary innovation. This conjecture is supported by the widespread phenomenon of TE exonization, a major driver of transcriptome diversification^54,55^, particularly in biological contexts where epigenetic control is relaxed, such as early mammalian development or tumorigenesis^56,57^. Although large-scale demethylation events are probably uncommon in nature, environmental stress or inefficient maintenance of methylation could transiently activate these normally repressed sequences in invertebrates.

In sum, our results demonstrate that DNA methylation can serve as a vehicle for intergenerational epigenetic inheritance in invertebrates, a long-standing hypothesis often invoked but rarely demonstrated with mechanistic detail. However, the primary function of gbM appears to be genome defence and be downstream of transcription, rather than the fine-tuning of host gene expression in response to environmental changes. Our results provide a cautionary perspective on the evolutionary and functional significance of DNA methylation in non-vertebrate animals.

## Method

This research was approved by the Queen Mary University of London ethics board.

### Animal husbandry and lines

*Nematostella* individuals from the laboratory-reared line were kindly provided by V. Modepalli. Juveniles and adults were housed in glass dishes containing 15‰ artificial sea water at 19 °C and fed freshly hatched *Artemia* nauplii three to five times a week, with weekly water changes. Spawning was induced following previously established protocols^58,59^. The gelatinous mass surrounding the eggs was removed using 4% L-cysteine (Sigma-Aldrich, no. 1.02838), after which eggs were washed and fertilized. Embryos were cultured in six-well plates at 22 °C for consistent developmental staging. Primary polyps metamorphosed were initially fed mashed nauplii until they can ingest intact nauplii and then transferred to larger glass dishes for further growth and maturation.

### DNA methylation inhibition

Following fertilization, zygotes were treated with different concentrations of cytidine analogues, including 5-azacytidine (Abcam, no. ab142744), zebularine (Abcam, no. ab141264) and decitabine (Abcam, no. ab120842), using DMSO as a control. Embryos were allowed to develop in solutions for 25 h, after which drugs were washed off. Treated embryos were then either sampled for DNA exaction or allowed to develop to polyp stage for survival assessment. Different treating windows were tested using 5 μM GSK-3484862 (MedChemExpress, no. HY-135146), with exposure durations ranging from 5 hours to 7 days. Several concentrations of GSK-3484862 were tested to optimize methylation depletion while maintaining solubility. The final concentration used (5 μM) corresponded to the highest fully soluble dose under our experimental conditions (0.1% DMSO, 15‰ artificial sea water, 22 °C), ensuring maximal efficacy without precipitation. Polyps at 8 dpf were collected for global DNA methylation measurement. For multi-omic profiling, zygotes produced by pooled gametes of several males and females were split into two wells containing either 5 μM GSK or equivalent amount of DMSO (0.1%) as the control. Three independent biological replicates for both treatment and control were prepared using different broodstocks. After 25 h, gastrula-stage embryos were washed and harvested for DNA, RNA and nucleus extraction.

To genetically induce demethylation, translation-blocking MO antisense oligonucleotides targeting DNMT1, DNMT3 and UHRF1 were used. MO sequences were designed and synthesized by Gene Tools as follows (5’–3’): DNMT1, TTCTTCGCAGGCAACCATTTCGTAG; DNMT3, CATGGTTGCCGACAGAGAGATCTA; UHRF1, CAAATGTTCGCACTTGAATCCACAT; and control MO, CCTCTTACCTCAGTTACAATTTATA. A 1-mM stock solution of each MO was prepared in nuclease-free water. De-jellied eggs were microinjected with 800 μM of a single MO or 400 μM each when two MOs were co-injected, alongside 0.2 mg ml^−1^ fluorescent dextran as a tracer (Thermo Scientific, no. D22910), using a FemtoJet 4i microinjector (Eppendorf). Injected eggs were fertilized within 3 h of spawning and embryos were allowed to develop until sampling for DNA extraction.

### Regeneration and growth series

The methylome profile of the regeneration series was compared between GSK-treated (demethylated) and control individuals. Each 8-month-old polyp was bisected and the oral half (tentacles, pharynx and mesentery) was allowed to regenerate a new aboral end (mesentery and physa) over ~3 weeks. This regenerated animal was then bisected again, this time with the newly formed aboral region allowed to regenerate, whereas tentacle and pharynx tissues from the original oral end were sampled. In the next cycle, physa from the aboral was sampled once regeneration of a new oral end was completed. We repeated bisections with alternating aboral/oral regeneration for a total of five rounds and collected newly regenerated tissues (excluding mesentery to avoid confounding gamete signal) at each cycle.

Global methylation during growth and maturation was also recorded in selected groups of animals. Whole animals were collected before 3 months of age, after which physa tissue was collected from one individual per group. At 12 mpf, one male from each group was induced to spawn and sperm was collected from three consecutive spawnings at 2-week intervals. Eggs from three spawnings (3-week intervals) of the same female in each group were pooled for DNA extraction as a result of low DNA yield from egg samples. To compare the DNA methylation amount between sperm and eggs within each group, gametes from several individuals (two to five females and five to seven males) were induced and collected for DNA extraction.

### F_1_ crossings

DNA methylation amounts of gametes from demethylated F_0_ individuals were validated with Oxford Nanopore (see below) before their use in any crossing. Methylation amounts of demethylated sperm were measured from excess sperm after their use for fertilization, whereas that of demethylated eggs were defined on the basis of prior measurements from the same female. After reciprocal crossing between treated and untreated F_0_ animals, the resulting F_1_ offspring were sampled at either gastrula or polyp stage for methylation analysis. For haplotype phasing, a single 4-month-old F_1_ juvenile derived from the cross between a demethylated F_0_ male and an untreated F_0_ female were collected for DNA extraction.

Two independent GSK-F_1_ lines were generated by crossing a pair of selected low-methylated GSK-F_0_ parents for each line. Similarly, we also generated two MO-F_1_ lines from DNMT1 + UHRF1 MO-injected F_0_ parents. For multi-omic profiling, F_1_ gastrulae embryos from these four lines were sampled at 25 hpf for DNA, RNA and nucleus extraction.

### DNA extraction

Genomic DNA (gDNA) from *Nematostella* embryos, polyps and tissues were isolated using Monarch Genomic DNA Purification Kit (New England Biolabs, no. T1030) following animal tissue lysis protocol, whereas sperm was processed using cell lysis protocol. For egg samples, because of their low gDNA/high lipid, protein and mitochondrion content, gDNA was purified by phenol-chloroform following mitochondrial depletion. To deplete mitochondria, gelatinous egg mass was first de-jellied and washed to remove nematosomes. Then, the oocyte nuclei were isolated by cytoplasm lysis and centrifugation as described in the ATAC-seq procedure (see below), depleting mitochondrial DNA. The prepared nuclei were digested using the Monarch kit cell lysis protocol including an RNA removal step. Crude gDNA was subsequently purified by standard phenol-chloroform extraction protocol and dissolved in nuclease-free water.

### Oxford Nanopore sequencing and methylation calling

For low passage DNA methylation profiling^33^, 30–100 ng of gDNA per samples was used to prepare multiplexed libraries by the Rapid Barcoding Kit V14 (SQK-RBK114), followed by sequencing on R10.4.1 chemistry flow cells using either MinION or PromethION platforms. For oocytes, we multiplexed libraries with the Native Barcoding Kit V14 (SQK-NBD114), where the ligation step reduces capture of the circular mitochondrial genome. Raw fast5 files were basecalled by Guppy (6.3.8) using the dna_r10.4.1_e8.2_400bps_modbases_5mc_cg_sup model and aligned to the reference genome GCF_932526225.1 (ref. ^60^) with minimap2 (2.24-r1122). We used modbam2bed (0.9.4) to convert modified base and mapped bam files to bed files, from which CpG methylation percentages were calculated. Reads mapping to the mitochondrial genome served as an internal negative control for methylation amount (Supplementary Table 2).

For high coverage Oxford Nanopore Technologies (ONT) sequencing, we first sheared gDNA to ~10 kilobases (kb) with a g-Tube (Covaris, no. 520079) and then used 1 μg as input for the SQK-LSK114 ligation protocol following manufacturer instructions. Libraries were sequenced in a PromethION flow cell (R10.4.1 chemistry) until reaching desired coverage (>30×). The resulting pod5 files were then used as input for Dorado (0.7.2), calling methylation with the 5mCG_5hmCG sup model while mapping it to the reference genome. Methylation calls were then extracted using Modkit pileup function (v.0.5.0), with the preset –cpg option.

### Enzymatic methyl-seq library preparation and analysis

We used 100 ng of gDNA, spiked with unmethylated phage lambda DNA and pUC19 CpG-methylated plasmid and sheared it to ~400 bp with a Covaris M220 Focused-ultrasonicator. We then constructed EM-seq libraries using the NEBNext Enzymatic Methyl-seq Kit (NEB, no. E7120) following manufacturer instructions. The resulting libraries were sequenced on an Illumina NovaSeq X Plus platform.

The EM-seq converted reads were mapped to the reference genome (GCF_932526225.1)^60^ using BS-Seeker2 with bowtie2 as a backend aligner^61^. Overlapping read pairs were merged using BBtools (bbmerge.sh) and treated as single-end reads. The resulting bam files were deduplicated using Sambamba and methylation calling was obtained using CGmapTools^62^. Non-conversion and enzymatic protection rates were assessed from the lambda and pUC19 controls, respectively (Supplementary Table 3). CGmap files were subset for CpG positions and converted to bigwig using the UCSC tool BedGraph2BigWig. These bigwig files were then uploaded into an Integrative Genome Browser session and used as input for DeepTools2 input to visualize methylation patterns^63^.

Publicly available WGBS and EM-seq datasets were downloaded from the National Center for Biotechnology Information (NCBI) and re-analysed using the same pipeline^22,28,39^. DeepTools2 multiBigwigSummary and plotCorrelation functions were used to cluster these datasets.

For quantitative analysis, CGmap files were read into R using the bsseq package, collapsing strand information for each CpG. These data were then used to compute global methylation amounts (total C calls/total coverage), weighted averages across genomic regions, including gene bodies or ATAC-seq peaks. Gene bodies were classified as methylated if their weighted methylation was ≥10% mCG, a permissive threshold chosen to capture genes exhibiting gbM. For ATAC-seq peaks, we applied a more stringent threshold of ≥20% mCG to confidently identify peaks originating from methylated regions and to avoid misclassification of peaks spanning boundaries between methylated and unmethylated DNA. Also, CpGs with coverage ≥10× were obtained for each EM-seq sample and means were computed for a given condition (for example, F_0_ GSK and F_0_ DMSO). The per CpG comparison was then plotted for all CpGs that had >0% mCG signal, excluding all the constitutively hypomethylated sites. DMRs were computed using the DSS R package (v.2.54.0) with the DMLtest() function with smoothing = TRUE and then the callDMR() function with delta = 0.5.

### ATAC-seq preparation and analysis

Nuclei and ATAC-seq libraries were prepared following a published protocol with modifications^64^. Briefly, fresh nuclei were dissociated from ~100 gastrulae embryos at 25 hpf by gentle homogenization with a pestle in lysis buffer. The nuclei suspension was passed through a 40-μm cell strainer (pluriSelect, no. 43-10040), washed, centrifuged and then counted with a haemocytometer. Approximately 5,000 nuclei were used per tagmentation reaction. Transposition was performed with in-house purified Tn5 transposase for 30 min at 37 °C. Following DNA clean-up, ATAC libraries were PCR amplified, size-selected using AMPure XP beads with double-sided selection and quality-checked on a TapeStation system (Agilent). The resulting libraries were sequenced with 2 × 150 bp reads on an Illumina NovaSeq X Plus platform.

Reads were then mapped to the reference genome using Chromap with ATAC-seq mode (--preset atac), which trims adaptors, shifts the insertion sites and cleans up duplicates automatically^65^. The resulting BEDPE files were used to compute the insertion distributions shown in Extended Data Fig. 4a to validate the quality of the assay, as well as fraction-of-reads-in-peaks scores >0.3 (Supplementary Table 4). Insertion site bigwigs were obtained using DeepTools2 bamCoverage function, normalizing using counts per million (CPM). These bigwigs were visualized using IGV and DeepTools2.

To compute differential ATAC-seq peak accessibility, we called peaks with MACS3 (-g 2.7e8 -q 0.05 -B)^66^. All peaks were merged using BEDTOOLS merge function and insert sites were counted using the featureCounts function in the SUBREAD package, requiring 20% read overlap (--fracOverlap 0.2). Insert counts per peak were read into R and normalized using DEseq2, which was then used to compute differential accessibility statistics using an FDR < 0.01. These normalized counts were also used to compare accessibility across samples.

Peaks were overlapped with genomic features obtained using the Bioconductor GenomicFeatures package, defining promoters as upstream 500 bp and downstream 200 bp from the transcriptional start site of each transcript.

TEs coordinates were obtained using the Earl Grey pipeline^67^, combining a de novo repeat detection with the RepBase annotation available for *Nematostella*, originally obtained from a previous fragmented version of the genome^32^. This combined library was then used to obtain Kimura distances against the consensus TE models.

Transcription-factor binding-site motif enrichment on peaks was obtained using Homer2, using the vertebrate motifs as reference. The motif footprinting was obtained using TOBIAS^68^, computing footprints on each ATAC-seq replicate with the ATACorrect and FootprintScores functions. Then, differential footprinting was computed for each combination of DMSO versus GSK replicates, only on statistically significant differentially accessible peaks. These results were read into R and sorted by *P*-value, filtering out those with *P* > 0.001. The motifs that appeared in at least four comparison combinations (DMSO replicate versus GSK replicate) were kept and then ranked for the median footprint score difference across all replicate comparisons.

### RNA-seq library preparation and analysis

Total RNA was extracted from gastrulae using the Monarch Total RNA Miniprep Kit (NEB, no. T2010S), followed by polyA selection with NEBNext Poly(A) mRNA Magnetic Isolation Module (NEB, no. E7490). RNA-seq libraries were then prepared from 800 ng of RNA using the NEBNext Ultra II Directional RNA Library Prep Kit for Illumina (NEB, no. E7760), according to the manufacturer’s instructions and sequenced on an Illumina NovaSeq X Plus platform to a depth of approximately 50 million paired-end reads per sample (Supplementary Table 5).

These data were mapped to the genome using HISAT2^69^, restricting maximum intron size to 40 kb and activating the transcriptome assembly flag (--max-intronlen 40000 --dta). The resulting bam files were processed with TElocal^70^ to obtain read counts on both protein coding genes and TE insertions, which were subsequently used for computing differential expression analysis with DEseq2. The bam files were converted into bigwig files using DeepTools bamCoverage function, using CPM for normalization. Each bam file was also used as input for StringTie requiring strand-specific information^71^, computing a de novo transcriptome for each replicate, then merged with the StringTie merge function to produce unique transcriptomes for GSK and DMSO samples. In parallel, short reads were mapped to the reference transcriptome with Kallisto^72^. The estimated counts values were then read into R, rounded and normalized with DEseq2. Differential expression analysis was then performed in F_0_ DMSO versus F_0_ GSK, F_1_ GSK or F_1_ MO pairs, using a FDR < 0.01. Gene ontology enrichments were calculated using TopGO R package (Supplementary Table 1). Gene ages were computed using OrthoFinder with select cnidarians and other metazoans, establishing the oldest possible clade as ‘Metazoa’ if an orthogroup was shared with sponge genomes, with all gene ages available in Supplementary Table 6.

Publicly available data were downloaded from NCBI^35,73^ and mapped against the reference transcriptome using Kallisto, collapsing isoform values into genes. Transcripts per million values were used to plot the transcription of DNA methylation machinery across development. To compare general transcriptional dynamics, the estimated counts were obtained for all replicates, normalized with DEseq2 and then standardized by defining the maximal expression value of each transcript as 1.

### CAGE-seq

The same RNA used to perform RNA-seq of 2 replicates of GSK-treated gastrulae was used for CAGE-seq library preparation, starting from 4 μg of input RNA. We followed previously described protocols^41^. Briefly, adaptor ligation and reverse transcription were followed by PCR amplification to incorporate adaptors and indexes. Libraries were then purified, quality-checked and sequenced on an Illumina NextSeq 2000 with the flow cell P4-50 kit, generating 70 bp single-end reads, obtaining more than 50 million single reads per sample (Supplementary Table 7).

Following sequencing, raw reads were demultiplexed using standard Illumina processing pipelines. Adaptor sequences and low-quality reads were trimmed and filtered using Trim Galore! (v.0.6.7, Babraham Bioinformatics) with default settings, after which the G added during the reverse transcription step was removed with cutadapt (v.4.6). Quality-filtered reads were aligned to the reference genome using STAR (v.5.1.0), ensuring that the 5′ end of each read was correctly mapped to its genomic location. Alignments were filtered to retain only primary, uniquely mapped reads with a mapping quality greater than 20.

Mapped data were processed using the CAGEr (v.2.14.0, Bioconductor; ref. ^74^) pipeline, following the recommended workflow. Signal normalization was performed using power-law normalization and TSS clusters were identified with a clustering threshold of 20 bp. Clusters were further filtered to remove artefacts and low-expression peaks and genomic coordinates were converted to a standardized format for further analyses.

### Histone modification and differential methylation analysis

The reads from an original ChIP–seq study profiling histone marks and cofactors along three developmental stages were downloaded from NCBI (GSE46488)^40^ and remapped to the new reference genome using bowtie2, allowing a maximum insert size of 2,000 bp (-X 2000) for paired-end reads. The resulting bam files were converted to bigwig using bamCoverage, removing PCR duplicates and normalizing using CPM. For an adult iChIP–seq dataset spanning both active and repressive histone modifications, we downloaded the normalized log_2_ signal-to-input bigwig tracks from the publication source (GSE329959)^42^, as these had been mapped to the same reference genome. These bigwig files were visualized on relevant coordinates using DeepTools2 or IGV.

We used the bigWigAverageOverBed tool from UCSC (https://hgdownload.soe.ucsc.edu/admin/exe/) to calculate the average histone modification signal for each DMR. These values were then used as input features to fit a linear regression model describing the methylation recovery at DMRs using the lm() function from R. Feature importance was calculated using the calc.relimp() function with the lmg metric from the relaimpo R package (v.2.2.7), with importance scores normalized so that they sum to 100%.

### Per-read methylation and haplotype phasing analysis

Modified base calling was performed using Dorado (v.0.7.2+9ac85c6) with the base-calling model sup,5mCG_5hmCG and base-modification calls were subsequently extracted with modkit (v.0.4.1) using the flags --pass-only and --motif CG 0. Base-modification calls were then filtered for calls where the base-quality score was at least 20. Custom R scripts were used to calculate the proportion of methylated CpG sites overlapping DMRs on a per-read basis.

Variants were called using Clair3 (v.1.0.10)^75^ and phased using WhatsHap phase (v.1.7)^76^. Variants identified in the juvenile offspring resulting from the cross between a GSK-treated male and an untreated female were assigned to parental haplotypes on the basis of informative sites: loci that were homozygous in the male parent (assessed from the sperm sample) but heterozygous in the offspring. At such sites, the paternal allele could be unambiguously assigned, whereas the alternate allele absent in the male parent must have been maternally inherited. The VariantAnnotation R package (v.1.52.0) was used to generate a VCF file containing these pedigree-phased variants, which was subsequently used to phase reads with WhatsHap haplotag.

### Reporting summary

Further information on research design is available in the Nature Portfolio Reporting Summary linked to this article.

## Supplementary information

Reporting Summary

Peer Review File

Supplementary Tables Supplementary Tables 1–7.

## Data Availability

All the sequencing data in this study have been uploaded to GEO (GSE307384). The genomic tracks can be visualized in the following IGV session: https://tinyurl.com/4e38zrbe.

## Code availability

The code used for the analysis in this paper is available via GitHub at https://github.com/AlexdeMendoza/Nematostella_GbM/ and via Zenodo at 10.5281/zenodo.19667318 (ref. ^77^).
