# Supplementary material for: Gene body methylation suppresses intragenic transcription and permits epigenetic inheritance in a cnidarian
Source: Nat Ecol Evol. 2026 Jun 2;10(7):1370–83. doi: 10.1038/s41559-026-03090-6 (PMC13345969; doi:10.1038/s41559-026-03090-6)
Supplement: Supplementary file 1 — Reporting Summary [file 41559_2026_3090_MOESM1_ESM.pdf]

nature portfolio

Corresponding author(s): Alex de Mendoza
Last updated by author(s): Apr 14, 2026

Reporting Summary

Nature Portfolio wishes to improve the reproducibility of the work that we publish. This form provides structure for consistency and transparency in reporting. For further information on Nature Portfolio policies, see our Editorial Policies and the Editorial Policy Checklist.

Statistics

For all statistical analyses, confirm that the following items are present in the figure legend, table legend, main text, or Methods section.

n/a	Confirmed	
☐	☒	The exact sample size (*n*) for each experimental group/condition, given as a discrete number and unit of measurement
☐	☒	A statement on whether measurements were taken from distinct samples or whether the same sample was measured repeatedly
☐	☒	The statistical test(s) used AND whether they are one- or two-sided<br>*Only common tests should be described solely by name; describe more complex techniques in the Methods section.*
☐	☒	A description of all covariates tested
☒	☐	A description of any assumptions or corrections, such as tests of normality and adjustment for multiple comparisons
☐	☒	A full description of the statistical parameters including central tendency (e.g. means) or other basic estimates (e.g. regression coefficient) AND variation (e.g. standard deviation) or associated estimates of uncertainty (e.g. confidence intervals)
☐	☒	For null hypothesis testing, the test statistic (e.g. *F*, *t*, *r*) with confidence intervals, effect sizes, degrees of freedom and *P* value noted<br>*Give P values as exact values whenever suitable.*
☒	☐	For Bayesian analysis, information on the choice of priors and Markov chain Monte Carlo settings
☒	☐	For hierarchical and complex designs, identification of the appropriate level for tests and full reporting of outcomes
☐	☒	Estimates of effect sizes (e.g. Cohen's *d*, Pearson's *r*), indicating how they were calculated

Our web collection on statistics for biologists contains articles on many of the points above.

Software and code

Policy information about availability of computer code

Data collection	Nanopore MinKNOW app was used to sequence the Nanopore libraries. This is the constantly updated version of Oxford Nanopore available to all users.
Data analysis	For Nanopore sequencing, base calling and methylation analysis Guppy v6.5.7, modbam2bed (0.9.1), Dorado, Modkit, BEDTools, UCSC bedGraphToBigWig, DeepTools2 and Bioconductor bsseq package for R were used. The SNPs and haplotypes were obtained using Clair3 and WhatsHap.<br>For RNA-seq analysis HISAT2, Stringtie. TElocal, and Kallisto were used to obtain counts, and DEseq2 for differential expression.<br>For ChIP-seq analysis fastp, bowtie2, Sambamba, and DeepTools2 were used.<br>For ATAC-seq, we used chromap for mapping.<br>For repeat annotation Earl Grey, RepeatModeler2 and RepeatMasker were used.<br>Version numbers of software/tools/algorithms/packages are reported in the Github repository: https://github.com/AlexdeMendoza/Nematostella_GbM/<br>The code used for the analysis of this paper can be found in: https://github.com/AlexdeMendoza/Nematostella_GbM/

For manuscripts utilizing custom algorithms or software that are central to the research but not yet described in published literature, software must be made available to editors and reviewers. We strongly encourage code deposition in a community repository (e.g. GitHub). See the Nature Portfolio guidelines for submitting code & software for further information.

Data

Policy information about availability of data

All manuscripts must include a data availability statement. This statement should provide the following information, where applicable:

- Accession codes, unique identifiers, or web links for publicly available datasets
- A description of any restrictions on data availability
- For clinical datasets or third party data, please ensure that the statement adheres to our policy

All the sequencing data in this study has been uploaded to GEO (GSE307384). Reviewer access token ajmtssokbbunzmz

Research involving human participants, their data, or biological material

Policy information about studies with human participants or human data. See also policy information about sex, gender (identity/presentation), and sexual orientation and race, ethnicity and racism.

Reporting on sex and gender	This study does not include human data.
Reporting on race, ethnicity, or other socially relevant groupings	*Please specify the socially constructed or socially relevant categorization variable(s) used in your manuscript and explain why they were used. Please note that such variables should not be used as proxies for other socially constructed/relevant variables (for example, race or ethnicity should not be used as a proxy for socioeconomic status).*<br>*Provide clear definitions of the relevant terms used, how they were provided (by the participants/respondents, the researchers, or third parties), and the method(s) used to classify people into the different categories (e.g. self-report, census or administrative data, social media data, etc.)*<br>*Please provide details about how you controlled for confounding variables in your analyses.*
Population characteristics	*Describe the covariate-relevant population characteristics of the human research participants (e.g. age, genotypic information, past and current diagnosis and treatment categories). If you filled out the behavioural & social sciences study design questions and have nothing to add here, write "See above."*
Recruitment	*Describe how participants were recruited. Outline any potential self-selection bias or other biases that may be present and how these are likely to impact results.*
Ethics oversight	*Identify the organization(s) that approved the study protocol.*

Note that full information on the approval of the study protocol must also be provided in the manuscript.

Field-specific reporting

Please select the one below that is the best fit for your research. If you are not sure, read the appropriate sections before making your selection.

☒ Life sciences ☐ Behavioural & social sciences ☐ Ecological, evolutionary & environmental sciences

For a reference copy of the document with all sections, see nature.com/documents/nr-reporting-summary-flat.pdf

Life sciences study design

All studies must disclose on these points even when the disclosure is negative.

Sample size	All measurements were obtained at least in duplicates and triplicates. These are independent animal crossings, therefore biological replicates.
Data exclusions	No data exclusion.
Replication	We performed quality control measures in all datasets, testing for correlation and congruence across replicates. Evidence for this is present throughout the manuscript.
Randomization	Not relevant to the study, these are treatment vs control, and the effect size (e.g. DNA methylation) was always tested, which makes the randomization unnecessary, as we were validating the effect size.
Blinding	No blinding required.

Reporting for specific materials, systems and methods

We require information from authors about some types of materials, experimental systems and methods used in many studies. Here, indicate whether each material, system or method listed is relevant to your study. If you are not sure if a list item applies to your research, read the appropriate section before selecting a response.

Materials & experimental systems

n/a	Involved in the study
☒	☐ Antibodies
☒	☐ Eukaryotic cell lines
☒	☐ Palaeontology and archaeology
☐	☒ Animals and other organisms
☒	☐ Clinical data
☒	☐ Dual use research of concern
☒	☐ Plants

Methods

n/a	Involved in the study
☒	☐ ChIP-seq
☒	☐ Flow cytometry
☒	☐ MRI-based neuroimaging

Animals and other research organisms

Policy information about studies involving animals; ARRIVE guidelines recommended for reporting animal research, and Sex and Gender in Research

Laboratory animals	Nematostella vectensis, the laboratory line that is kept at most european laboratories.
Wild animals	None.
Reporting on sex	When sampling adults, they were sexed. Embryos and juveniles cannot be sexed in Nematostella.
Field-collected samples	None.
Ethics oversight	This research was approved by the Queen Mary University of London ethics board.

Note that full information on the approval of the study protocol must also be provided in the manuscript.

Plants

Seed stocks	No plants used
Novel plant genotypes	N/A
Authentication	N/A
