# Supplementary material for: Gene body methylation suppresses intragenic transcription and permits epigenetic inheritance in a cnidarian
Source: Nat Ecol Evol. 2026 Jun 2;10(7):1370–83. doi: 10.1038/s41559-026-03090-6 (PMC13345969; doi:10.1038/s41559-026-03090-6)
Supplement: Supplementary file 2 — Peer Review File [file 41559_2026_3090_MOESM2_ESM.pdf]

nature portfolio

Peer Review File

The role and inheritance of gene body methylation in a cnidarian

Corresponding Author: Dr Alex de Mendoza

Version 0:

Decision Letter:

30th January 2026

*Please ensure you delete the link to your author homepage in this e-mail if you wish to forward it to your co-authors.

Dear Alex,

Your manuscript entitled "The role and inheritance of gene body methylation in an invertebrate" has now been seen by two reviewers, whose comments are attached. The reviewers have raised a number of concerns which will need to be addressed before we can offer publication in Nature Ecology & Evolution. We will therefore need to see your responses to the criticisms raised and to some editorial concerns, along with a revised manuscript, before we can reach a final decision regarding publication.

In addition to the reviewers' comments, we would like you to tweak the abstract to give a little more evolutionary flavour to it.

We therefore invite you to revise your manuscript taking into account all reviewer and editor comments. Please highlight all changes in the manuscript text file in Microsoft Word format.

We are committed to providing a fair and constructive peer-review process. Do not hesitate to contact us if there are specific requests from the reviewers that you believe are technically impossible or unlikely to yield a meaningful outcome.

When revising your manuscript:

* Include a “Response to reviewers” document detailing, point-by-point, how you addressed each reviewer comment. If no action was taken to address a point, you must provide a compelling argument. This response will be sent back to the reviewers along with the revised manuscript.

* If you have not done so already please begin to revise your manuscript so that it conforms to our Article format instructions at http://www.nature.com/natecolevol/info/final-submission. Refer also to any guidelines provided in this letter.

* Include a revised version of any required reporting checklist. It will be available to referees (and, potentially, statisticians) to aid in their evaluation if the manuscript goes back for peer review. A revised checklist is essential for re-review of the paper.

* Extended Data Figures - please ensure that any supplementary figures and tables that are crucial to the manuscript’s conclusions are converted into Extended Data figures and tables to increase visibility of these data. Extended Data figures and tables are online-only (present in the online PDF and full-text HTML versions of the paper), peer-reviewed display items that provide essential background to the article but are not included in the main article due to space constraints. A maximum of ten Extended Data display items (figures and tables) is permitted.

Please use the link below to submit your revised manuscript and related files:

Link Redacted

Note: This URL links to your confidential home page and associated information about manuscripts you may have submitted, or that you are reviewing for us. If you wish to forward this email to co-authors, please delete the link to your homepage.

We hope to receive your revised manuscript within four to eight weeks. If you cannot send it within this time, please let us

know. We will be happy to consider your revision so long as nothing similar has been accepted for publication at Nature Ecology & Evolution or published elsewhere.

Nature Ecology & Evolution is committed to improving transparency in authorship. As part of our efforts in this direction, we are now requesting that all authors identified as 'corresponding author' on published papers create and link their Open Researcher and Contributor Identifier (ORCID) with their account on the Manuscript Tracking System (MTS), prior to acceptance. ORCID helps the scientific community achieve unambiguous attribution of all scholarly contributions. You can create and link your ORCID from the home page of the MTS by clicking on 'Modify my Springer Nature account'. For more information please visit please visit www.springernature.com/orcid.

Please do not hesitate to contact me if you have any questions or would like to discuss these revisions further.

We look forward to seeing the revised manuscript and thank you for the opportunity to review your work.

[redacted]

Reviewer expertise:

Reviewer #1: Epigenetic regulation of gene expression and embryonic development

Reviewer #2: Evolution of chromatin modifications, cnidarians

Reviewers' comments:

Reviewer #1 (Remarks to the Author):

In this manuscript, the authors use Nematostella as a model to investigate the effects of 5mC depletion. They found that reducing 5mC level leads to altered chromatin accessibility and aberrant transcription initiation, while it has limited influence on development. Interestingly, the altered 5mC landscape is partially recovered in germline, then inherited by the next generation without reprogramming. Overall, this paper is well presented and expands our knowledge on the function of gene body DNA methylation in invertebrates.
However, for following reasons, i think this work is not suitable for publication on Nature Ecology & Evolution.
Major:
1.The authors proposed that gbM suppresses TE transcription (line 28 to 31). However, although being less studied in invertebrates, 5mC mediated TE silencing has been extensively reported in recent years. (e.g. https://doi.org/10.1016/j.tig.2022.02.009; https://doi.org/10.1038/s41576-019-0106-6) This undermines the novelty of the authors' findings.
2.The study on 5mC recovery and inheritance did not provide enough novel insights. Firstly, the analysis between 5mC recovery and chromatin states in figure 3 remain correlative and lack mechanistic study. Secondly, it is already known that cnidarian lacks 5mC reprogramming. (e.g. https://doi.org/10.1093/nsr/nwz064) So it is not surprising to see the aberrant 5mC pattern to be passively inherited. In addition, Fig4.b shows that eggs from GSK treated female have similar 'partially recovered' 5mC level with sperm from GSK treated male, which is in conflict with the conclusion from extended data Fig7.
Minor:
1.The statistic works appears to be arbitrary. For example, in Fig.2 d and f, the authors use two different thresholds to classify methylation states of genes and peaks. The authors need to justify their selection on different thresholds.
2.The authors claim that non-genetic changes could be subject to natural selection (line 426). As loss of 5mC results in viable embryo with limited effects on gene expression (line 27 to 28), they need direct evidence linking the 5mC loss to a measurable impact on fitness to make this claim.

Reviewer #1 (Remarks on code availability):

I opened the link, but I can't see the code.

Reviewer #2 (Remarks to the Author):

The role of DNA methylation has been extensively studied; however, in animals, our understanding of its function is largely restricted to vertebrates. In invertebrates, methylation primarily occurs within gene bodies and is correlated with transcriptional activity. The role of this form of DNA methylation and its inheritance remain largely unexplored. Xu et al. address this problem using the sea anemone Nematostella vectensis as a model. By removing the majority of DNA methylation, they show that the primary role of gene body methylation is to suppress cryptic regulatory elements within genes. In addition, they demonstrate the absence of epigenetic reprogramming in Nematostella.

Overall, this is a truly excellent paper. The study is of extremely high quality, combining multiple lines of evidence and approaches to generate a very coherent story. In addition, the writing is excellent and the paper is a joy to read.

I am strongly in favour of publication of this manuscript and have just a few relatively minor comments that I think the authors should addres:

1. My only “major” comment is that the authors use an inhibitor to generate a hypomethylated state and study its effects. They do not, however, completely abolish DNA methylation (for example, by generating DNMT mutants), and so it is possible that some functions could still be achieved via the low level of remaining methylation. I think the authors should briefly discuss this limitation, without overemphasizing it, and note that generating mutants in the future would be important/interesting.

2. The authors do not show any tests used to determine the concentration of GSK applied. Including these data, if they exist, would be important.

3. Line 148: “prior to extensive cell-type diversification.” I am not sure this is fully accurate, as many major lineages are already present at this stage and there are even some relatively differentiated cell types (e.g., cnidocytes, neurons).

4. Line 159: The authors do not discuss the types of genes that are differentially expressed. It would be interesting to know whether these genes belong to particular functional classes, whether they are highly conserved, or Nematostella-specific, etc.

5. Line 198: The use of the term “modestly” is unclear. Does this mean that few genes were affected, or that the genes that were affected did not change greatly in their expression levels?

6. Line 209: I understand what the authors mean by a “quantitative” change here, as opposed to an on–off effect, but it might be worth expanding this slightly for readers who are less familiar with this distinction.

7. Lines 87 and 253: The authors use 5mCG instead of 5mC, which is the term used throughout the rest of the paper and in the figures.

8. When discussing the recovery of DNA methylation patterns, it is unclear to me why expression in early stages would correlate with later recovery, as opposed to genes highly expressed at later stages. Could the authors expand on this point?

Reviewer #2 (Remarks on code availability):

The code is actually not available on the link provided. The link does not work.

********************END********************

Version 1:

Decision Letter:

20th March 2026

Dear Alex,

Thank you for submitting your revised manuscript "The role and inheritance of gene body methylation in an invertebrate" (NATECOLEVOL-25124326A). It has now been seen again by the original reviewers and their comments are below. The reviewers find that the paper has improved in revision, and therefore we'll be happy in principle to publish it in Nature Ecology & Evolution, pending minor revisions to satisfy the reviewers' final requests and to comply with our editorial and formatting guidelines.

We are now performing detailed checks on your paper and will send you a checklist detailing our editorial and formatting requirements in about a week. Please do not upload the final materials and make any revisions until you receive this additional information from us.

Thank you again for your interest in Nature Ecology & Evolution. Please do not hesitate to contact me if you have any questions.

[redacted]

Reviewer #1 (Remarks to the Author):

The authors have provided a clear and convincing response to my major concern regarding the novelty of their work. Their rebuttal appropriately emphasises that the role of gene body methylation in transposable element repression has not been previously documented in invertebrates, where the prevailing paradigm links gbM primarily to actively transcribed genes. The revised abstract now appropriately frames their finding within this evolutionary and ecological context.

While DNA methylation is the focus here, many animals also employ small-RNA-mediated pathways, particularly PIWI–piRNA systems, as a conserved mechanism for transposable element repression; a brief comment on how gbM-mediated repression relates to or differs from piRNA-mediated control would help situate the findings in the broader landscape of TE regulatory mechanisms.
Based on the authors' responses and the revised statements, I believe this manuscript is now suitable for consideration for publication in Nature Ecology & Evolution.

Reviewer #2 (Remarks to the Author):

As I said in my original review I believe this manuscript to be of extremely high quality. In addition it addresses a broadly interesting question and provided an abundance of new data and understanding (within the limits of what can be expected in a single manuscript).

The authors have addressed all of my comments sufficiently. In addition, I believe the authors have also addressed the comments of the other reviewer. I therefore enthusiastically support the publication of this manuscript.

Reviewer #2 (Remarks on code availability):

I am now able to access the associated code and I have looked through briefly. I have not tested the individual code but in a general sense it all seems to be there.

Open Access This Peer Review File is licensed under a Creative Commons Attribution 4.0 International License, which permits use, sharing, adaptation, distribution and reproduction in any medium or format, as long as you give appropriate credit to the original author(s) and the source, provide a link to the Creative Commons license, and indicate if changes were made.
In cases where reviewers are anonymous, credit should be given to 'Anonymous Referee' and the source.
The images or other third party material in this Peer Review File are included in the article's Creative Commons license, unless indicated otherwise in a credit line to the material. If material is not included in the article's Creative Commons license and your intended use is not permitted by statutory regulation or exceeds the permitted use, you will need to obtain permission directly from the copyright holder.
To view a copy of this license, visit https://creativecommons.org/licenses/by/4.0/

Reviewer #1:

In this manuscript, the authors use Nematostella as a model to investigate the effects of 5mC depletion. They found that reducing 5mC level leads to altered chromatin accessibility and aberrant transcription initiation, while it has limited influence on development. Interestingly, the altered 5mC landscape is partially recovered in germline, then inherited by the next generation without reprogramming. Overall, this paper is well presented and expands our knowledge on the function of gene body DNA methylation in invertebrates. However, for following reasons, i think this work is not suitable for publication on Nature Ecology & Evolution.

Major:

1.The authors proposed that gbM suppresses TE transcription (line 28 to 31). However, although being less studied in invertebrates, 5mC mediated TE silencing has been extensively reported in recent years. (e.g. https://doi.org/10.1016/j.tig.2022.02.009; https://doi.org/10.1038/s41576-019-0106-6) This undermines the novelty of the authors' findings.

Response: We respectfully disagree that this undermines the novelty of our findings. The central question addressed in our study is not whether DNA methylation can silence transposable elements in general, but specifically what gene body methylation (gbM) does in invertebrates. Historically, gbM has not been viewed as a silencing mechanism in invertebrates. Instead, it has been widely associated with constitutively expressed genes, leading to the prevailing interpretation that gbM plays a regulatory role in host gene expression rather than in transposable element control. This interpretation has shaped a large body of ecological and evolutionary literature, where gbM has been proposed to mediate phenotypic plasticity, caste determination, and environmental adaptation. Indeed, even in plants, where gbM has been studied extensively, its functional role in gene regulation remains actively debated, with recent studies continuing to propose regulatory functions. In invertebrates, evidence linking gbM to transposable element suppression within gene bodies has been lacking. Our work addresses this gap by demonstrating experimentally that loss of gbM leads to aberrant transcriptional initiation from intragenic transposable elements and increased chromatin accessibility, establishing a causal role for gbM in maintaining transcriptional integrity (**Fig. 2e-g**).

While the role of DNA methylation in transposable element silencing is well established in vertebrates, vertebrate methylomes differ fundamentally from those of most animals. Vertebrates exhibit genome-wide hypermethylation, whereas the vast majority of animal phyla retain sparse methylation primarily restricted to gene bodies. How that transition from sparse methylation to hypermethylation occurred is hard to understand without having a clear idea of the function of gbM in invertebrates, the ancestral state. From our data, probably it was a response to TE expansion and control beyond gene bodies that triggered the vertebrate hypermethylated state.

2.The study on 5mC recovery and inheritance did not provide enough novel insights. Firstly, the analysis between 5mC recovery and chromatin states in figure 3 remain correlative and lack mechanistic study.

Response: We agree that our analysis identifies associations between chromatin features and methylation recovery, and that the precise molecular mechanisms linking these chromatin states to de novo methylation targeting remain to be fully elucidated. However, establishing these relationships already represents an important advance. Our study leverages the most comprehensive dataset of histone modifications currently available for any invertebrate with a functional DNA methylation system. This enables us, for the first time, to systematically identify the chromatin features that predict DNA methylation recovery following experimental depletion.

Specifically, we demonstrate that methylation recovery is strongly biased toward transcriptionally active gene bodies marked by H3K36me3 and H3K79me2/3 (previously unreported), while regions enriched in heterochromatic marks such as H3K9me3 show delayed or incomplete recovery (Fig. 3f-h). These findings provide key mechanistic insight into how DNA methylation targeting is guided in invertebrate genomes, and help explain why gene body methylation is preferentially associated with actively transcribed regions.

Importantly, we also identify localised heterochromatic regions that harbor DNA methylation (a unique observation in the invertebrate literature) but exhibit slow recovery kinetics (Fig. 3h). These regions are particularly prone to epigenetic variability across independent laboratory populations of *Nematostella* (Extended Data Fig. 9b), linking chromatin context, methylation

recovery dynamics, and epigenetic inheritance. To our knowledge, this is the first study to experimentally connect chromatin state with methylation recovery dynamics and epigenetic variability in an invertebrate system.
While dissecting the molecular recruitment mechanisms of DNMT1 & 3 will require future biochemical and genetic studies, the framework established here provides a critical foundation for understanding how methylation patterns are established, maintained, and inherited in animal genomes.

Secondly, it is already known that cnidarian lacks 5mC reprogramming. (e.g. https://doi.org/10.1093/nsr/nwz064) So it is not surprising to see the aberrant 5mC pattern to be passively inherited.

Response: We agree that previous studies have reported that cnidarians do not undergo genome-wide epigenetic reprogramming under steady-state conditions, and we explicitly acknowledge this in the manuscript. However, what has not been previously examined is how DNA methylation patterns are re-established following disruption, and whether recovery occurs uniformly across tissues, genomic regions, or generations.
Our study provides the first experimental characterization of methylation recovery dynamics in an invertebrate following global methylation depletion. We show that somatic tissues fail to restore methylation even after multiple rounds of whole body regeneration and extensive cell division (Fig. 3b), while the germline exhibits partial and selective recovery (Fig. 3d). This demonstrates that methylation restoration is not a passive consequence of DNA replication, but a regulated process linked to germline-specific de novo methylation activity.
Importantly, we also demonstrate that aberrant methylation states are inherited in a locus-specific manner, with some genomic regions recovering efficiently while others remain stably hypomethylated across generations (Fig. 3e). This reveals that epigenetic inheritance in cnidarians is not uniform, but constrained by chromatin context and transcriptional activity. Furthermore, by crossing individuals with distinct methylation states, we show that methylation recovery occurs independently on each parental haplotype (Fig. 4b and Extended Data Fig 9a), indicating the absence of a mechanism that restores a canonical methylation pattern using the homologous chromosome as a template, which has only been recently demonstrated in fish (Ross et al. 2023).
Finally, our perturbation-based approach allows us to disentangle maintenance and de novo methylation processes, revealing that transcriptional activity and associated chromatin features strongly influence methylation recovery. These findings provide direct mechanistic insight into how DNA methylation patterns are established and inherited in invertebrates, which could not be inferred from steady-state observations alone.

In addition, Fig4.b shows that eggs from GSK treated female have similar ‘partially recovered’ 5mC level with sperm from GSK treated male, which is in conflict with the conclusion from extended data Fig7.

Response: We apologize for the confusion. Methylation recovery levels vary between individuals and between successive spawnings, even within the same individual (see Fig. 3c,d). As a result, the exact levels of methylation observed in eggs and sperm can differ across experiments.

In Fig. 4b, we intentionally selected sperm samples that displayed the lowest methylation recovery among the treated individuals. This choice facilitated the detection of haplotype-specific methylation inheritance when crossing with wild-type gametes. Because of the natural variability between spawnings and individuals, sperm methylation levels can occasionally appear similar to, or even lower than, those of eggs in specific examples.

However, the overall trend across our datasets is that sperm recover methylation more rapidly than eggs. For instance, in Extended Data Fig. 9, sperm consistently show higher methylation levels than eggs within the same experimental groups. We have clarified this point in the manuscript text and in the legend of Fig. 4b to avoid confusion.

Minor:
1.The statistic works appears to be arbitrary. For example, in Fig.2 d and f, the authors use two different thresholds to classify methylation states of genes and peaks. The authors need to justify their selection on different thresholds.
Response: We thank the reviewer for raising this point. The use of different thresholds reflects the distinct genomic scales and biological interpretations of gene bodies versus ATAC-seq peaks.
Gene bodies are large genomic features, ranging from a few kilobases to over 100 kb. As a result, their average methylation levels represent the aggregate signal across many CpG sites, including regions of heterogeneous methylation. Our goal was to distinguish genes that are genuinely gene body methylated from those that are essentially unmethylated. We therefore used a relatively permissive threshold (>10% mCG), consistent with prior studies in invertebrates, to capture genes that clearly exhibit gene body methylation while minimizing false negatives.
In contrast, ATAC-seq peaks are much narrower features, typically on the order of hundreds to a few thousand base pairs. These peaks frequently occur near promoter regions, which are constitutively unmethylated, and may span transitions between methylated and unmethylated DNA. For this reason, we applied a more stringent threshold (>20% mCG) to confidently classify peaks as originating from methylated regions. This conservative threshold ensures that the "methylated peaks" we analyze represent bona fide accessibility gains within previously methylated DNA, rather than artifacts arising from boundary regions.
Importantly, our conclusions are robust to reasonable variation in these thresholds, and the distinction between methylated and unmethylated regions remains clearly supported by the data. We have now clarified the rationale for these thresholds in the Methods section.

To reassure the reviewer that our choice of thresholds does not affect the conclusions, we repeated the analysis using alternative cutoffs for defining methylated genes (5%, 15%, 20%, and 25% mCG), as in **Fig. 2d**. Across all thresholds tested, the results were consistent: the vast majority of methylated genes remained transcriptionally unchanged, most downregulated genes were methylated, and most upregulated genes were unmethylated. These findings demonstrate that our conclusions are robust to the specific methylation threshold applied (see figure below).

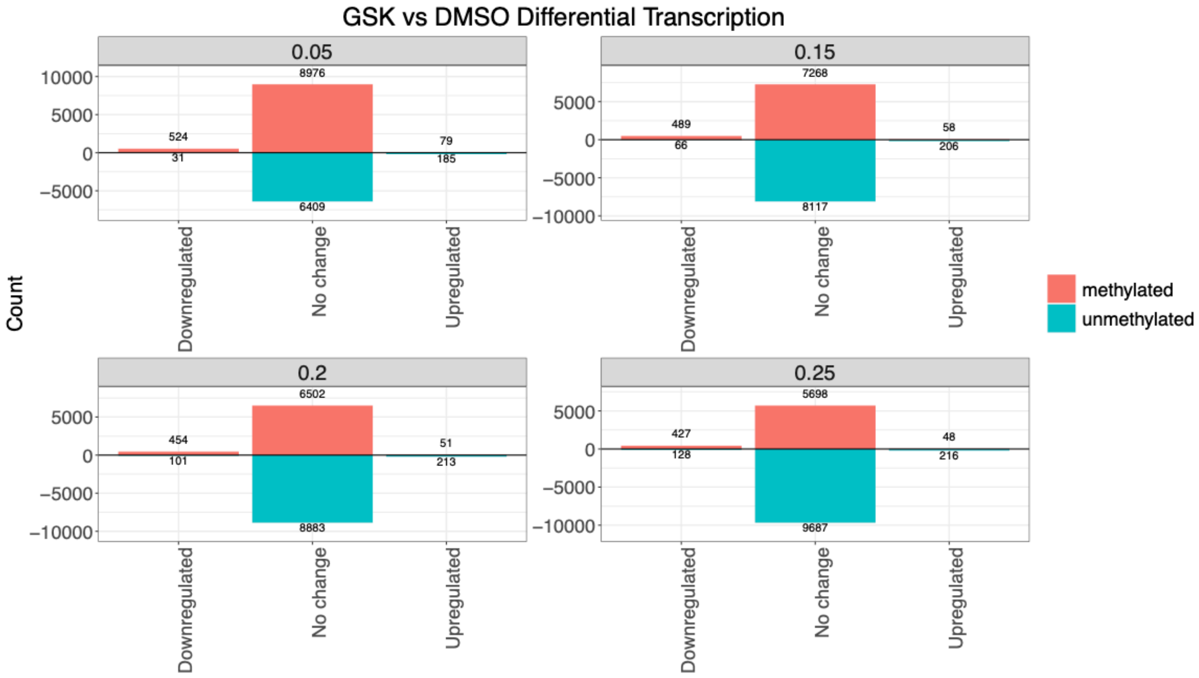

Figure 1. **Gene body methylation threshold**: Number of differentially expressed and stably expressed genes at the gastrula stage. Genes are classified as methylated using several thresholds highlighted on top of each panel, and are shown in red; unmethylated genes in blue.

2.The authors claim that non-genetic changes could be subject to natural selection (line 426). As loss of 5mC results in viable embryo with limited effects on gene expression (line 27 to 28), they need direct evidence linking the 5mC loss to a measurable impact on fitness to make this claim.

Response: We thank the reviewer for raising this important point. We agree that demonstrating direct fitness consequences of altered methylation would require dedicated long-term ecological or evolutionary experiments, which are beyond the scope of the present study. Our claim is not that we directly demonstrate selection acting on these epimutations, but that we identify heritable regulatory variation that could serve as a substrate for selection. Specifically, we show that loss of DNA methylation leads to stable, locus-specific changes in chromatin accessibility and the emergence of novel transcriptional start sites, often associated with transposable elements. These altered regulatory states can persist across generations due to incomplete methylation recovery. The generation of heritable variation in regulatory elements is widely recognized as a key source of evolutionary novelty, regardless of whether individual variants are neutral, deleterious, or advantageous.

Importantly, most epimutations we observe appear to have limited immediate impact on organismal viability, which is consistent with the idea that many regulatory changes may initially be neutral. However, such changes can still influence transcriptional architecture and genome stability, and therefore represent potential targets for selection under specific environmental or genetic contexts. The evolutionary literature on transposable elements provides numerous examples of regulatory sequences originating from previously silenced elements that were later co-opted into functional roles.

We have revised the manuscript, especially the abstract, to clarify that our findings demonstrate the existence of heritable epigenetic variation with regulatory potential, rather than directly demonstrating fitness effects.

Reviewer #1 (Remarks on code availability):

I opened the link, but I can't see the code.
Response: This was an oversight on our part, as the GitHub repository was not set to public visibility. The repository is now publicly accessible, and the code can be found at the following link: https://github.com/AlexdeMendoza/Nematostella_GbM/.

Reviewer #2 (Remarks to the Author):

The role of DNA methylation has been extensively studied; however, in animals, our understanding of its function is largely restricted to vertebrates. In invertebrates, methylation primarily occurs within gene bodies and is correlated with transcriptional activity. The role of this form of DNA methylation and its inheritance remain largely unexplored. Xu et al. address this problem using the sea anemone Nematostella vectensis as a model. By removing the majority of DNA methylation, they show that the primary role of gene body methylation is to suppress cryptic regulatory elements within genes. In addition, they demonstrate the absence of epigenetic reprogramming in Nematostella.

Overall, this is a truly excellent paper. The study is of extremely high quality, combining multiple lines of evidence and approaches to generate a very coherent story. In addition, the writing is excellent and the paper is a joy to read.

I am strongly in favour of publication of this manuscript and have just a few relatively minor comments that I think the authors should addres:

1. My only “major” comment is that the authors use an inhibitor to generate a hypomethylated state and study its effects. They do not, however, completely abolish DNA methylation (for example, by generating DNMT mutants), and so it is possible that some functions could still be achieved via the low level of remaining methylation. I think the authors should briefly discuss this limitation, without overemphasizing it, and note that generating mutants in the future would be important/interesting.

Response: We thank the reviewer for this thoughtful comment and agree that our approach does not completely eliminate DNA methylation. Our goal was to achieve widespread and specific depletion of 5mC while minimizing confounding effects associated with complete loss of DNMT proteins. DNMT1, in particular, is known to have non-catalytic functions independent of its methyltransferase activity. Therefore, full genetic knockout of DNMT1 could introduce additional phenotypes unrelated to DNA methylation loss per se.
To address this, we used both pharmacological inhibition and morpholino-mediated depletion to reduce methylation levels through complementary approaches. Importantly, our analysis of second-generation embryos demonstrates that many of the observed regulatory effects

persist in the absence of DNMT1 depletion, confirming that altered methylation states themselves, rather than loss of DNMT1 protein function, underlie these changes.
Probably generating catalytic mutants of DNMT enzymes would provide valuable additional insight. However, precise genome editing approaches such as base editing are not yet fully established in *Nematostella*, and complete elimination of methylation would likely require targeting both DNMT1 and DNMT3. We have now added the following text to the Discussion to acknowledge this limitation and outline future directions:
"*Although widespread methylation loss was achieved using both inhibitors and morpholinos, residual 5mC remained detectable. Complete elimination of DNA methylation would likely require genetic disruption of both DNMT1 and DNMT3. However, loss of DNMT proteins could introduce additional phenotypes unrelated to methylation, given their known non-catalytic roles. Future genetic approaches targeting catalytic activity specifically will help further dissect the functional consequences of total DNA methylation loss*."

2. The authors do not show any tests used to determine the concentration of GSK applied. Including these data, if they exist, would be important.

Response: We thank the reviewer for raising this point. We did test multiple concentrations of the GSK inhibitor prior to selecting the final experimental conditions. The primary limitation was the solubility of the compound, which is capped at approximately 5 µM under our experimental conditions (0.1% DMSO, 15‰ artificial sea water, 22°C). We tested concentrations from 5 µM to 20 µM, and observed strong precipitation in the medium during treatment starting from 10 µM. Therefore, we selected the highest concentration that remained fully soluble and did not produce precipitation, ensuring consistent delivery and maximal depletion of DNA methylation. For comparison, studies in mammalian cell lines have shown that concentrations as low as 0.2 µM are sufficient to inhibit DNMT1 activity, suggesting that the concentration used here is well within the effective range while not showing evidence of cytotoxicity (Pappalardi et al. 2021). We have now clarified this rationale in the Methods section. Specifically, we state:

"*Multiple concentrations of GSK-3484862 were tested to optimize methylation depletion while maintaining solubility. The final concentration used (5 µM) corresponded to the highest fully soluble dose under our experimental conditions (0.1% DMSO, 15‰ artificial sea water, 22°C), ensuring maximal efficacy without precipitation.*".

3. Line 148: "prior to extensive cell-type diversification." I am not sure this is fully accurate, as many major lineages are already present at this stage and there are even some relatively differentiated cell types (e.g., cnidocytes, neurons).

Response: We agree that several differentiated cell types, including neurons and cnidocytes, are already present at the gastrula stage. Our intent was to emphasize that the gastrula represents an intermediate developmental stage that is more transcriptionally and cellularly homogeneous than later stages such as planula or juvenile polyps, while still occurring after zygotic genome activation. To better reflect this, we have revised the sentence to avoid implying the absence of differentiated cell types. The text now reads:
"*To investigate the regulatory impact of DNA methylation loss, we focused on the gastrula stage (25 hpf), a timepoint when the correlation between gbM and transcription remains high*

(Supplementary Fig. 1d), while overall cell-type complexity is still lower than in later developmental stages."

4. Line 159: The authors do not discuss the types of genes that are differentially expressed. It would be interesting to know whether these genes belong to particular functional classes, whether they are highly conserved, or Nematostella-specific, etc.

Response: We thank the reviewer for this helpful suggestion. We had already performed Gene Ontology enrichment analysis and reported these results in Supplementary Table 1, but we agree that highlighting the functional categories in the main text improves clarity and interpretation. We have also included a gene age analysis, where "Metazoa" is the oldest possible category, but is enough to distinguish between old and newer stratums. We have now added the following description to the Results section:

"*Downregulated methylated genes were primarily enriched for functions related to amino acid metabolism, whereas upregulated unmethylated genes showed weaker functional enrichment, with a couple of genes associated with chromatin-related processes, including H3K4 methylation and CpG binding (**Supplementary Table 1**). Consistent with these differences, downregulated genes were enriched for older gene ages, while upregulated unmethylated genes were biased toward younger gene age classes (Extended Data Fig. 3d)*"

And added this new panel in **Extended Data Figure 3**:

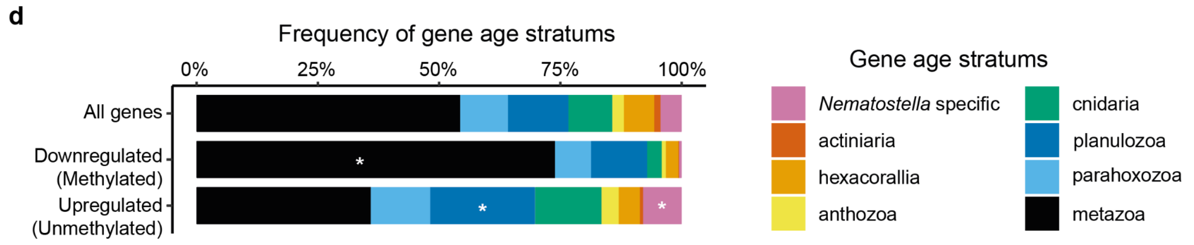

These results further support the conclusion that loss of gene body methylation does not broadly disrupt core developmental gene programs, but instead affects a limited subset of genes, consistent with the overall modest transcriptional impact observed. Also, it corroborates previous reports that methylated genes tend to have older origins than unmethylated genes, consistent with more "housekeeping" functions.

5. Line 198: The use of the term "modestly" is unclear. Does this mean that few genes were affected, or that the genes that were affected did not change greatly in their expression levels?

Response: Our intent was to indicate that relatively few genes were affected, rather than referring to the magnitude of expression changes. To clarify this, we have revised the sentence to explicitly state the proportion of affected genes. The text now reads:
"*Open methylated peaks were largely intronic and rarely affected gene expression: only 13% of genes harboring new peaks were differentially expressed (48 downregulated, 11 upregulated; Supplementary Fig. 4c).*"

6. Line 209: I understand what the authors mean by a “quantitative” change here, as opposed to an on–off effect, but it might be worth expanding this slightly for readers who are less familiar with this distinction.

Response: We have expanded the description to make the distinction explicit for a broader readership. The revised text now reads:
“*Only transposable elements that were originally silenced and methylated showed clear reactivation in an on–off manner upon methylation loss. In contrast, changes observed in transposable elements that were already transcriptionally active were generally quantitative, reflecting shifts in the level of transcription rather than de novo activation (Supplementary Fig. 5d).*”

7. Lines 87 and 253: The authors use 5mCG instead of 5mC, which is the term used throughout the rest of the paper and in the figures.

Response: We thank the reviewer for pointing out this inconsistency. We have now replaced all instances of “5mCG” with “5mC” to maintain consistent terminology throughout the manuscript and figures.

8. When discussing the recovery of DNA methylation patterns, it is unclear to me why expression in early stages would correlate with later recovery, as opposed to genes highly expressed at later stages. Could the authors expand on this point?

Response: Our inference is based on several independent observations. First, the correlation between gene body methylation and transcription is strongest during early development and progressively weakens at later stages (Extended Data Fig. 1d), suggesting that methylation patterns are more tightly linked to transcriptional activity during early developmental windows. Second, our regeneration experiments show that somatic tissues exhibit minimal methylation recovery even after extensive cell division, indicating limited de novo methylation activity in somatic contexts. In contrast, substantial recovery occurs during germline development and early embryogenesis. Notably, this is not tightly associated with DNMT3 transcriptional dynamics, as it is expressed high in early development, but also comes back in later development (Fig. 1c). Because DNMT3 recruitment is associated with transcription-coupled chromatin features such as H3K36me3 and H3K79me2/3, genes expressed during these periods are more likely to regain methylation. Why somatic DNMT3 fails to do the same remains a mystery, and would probably require developing precise antibodies to trace sub-cellular localisation.
This suggests that methylation recovery depends not simply on whether a gene is expressed, but on whether it is expressed during developmental windows when de novo methylation activity is highest. Genes activated later in development may therefore experience less efficient remethylation.

From an evolutionary perspective, we speculate that this stage-specific recovery may represent a strategy to prioritize genome integrity during germline and early embryonic development, when transposable element (TE) insertions are most likely to be propagated to the next generation. In contrast, TE activity in somatic tissues would have more limited evolutionary consequences, as somatic insertions are not inherited. Consistent with this idea,

many TEs are preferentially active during germline and early developmental stages, making these windows particularly vulnerable to genomic disruption.
This interpretation is also supported by our previous observations in other non-vertebrate bilaterians, where early developmental stages exhibit higher levels of gene body methylation compared to adult somatic tissues (Guynes et al. 2024). This pattern suggests that germline and early embryogenesis may act as periods of methylation replenishment, while somatic tissues may be more permissive to gradual methylation loss over time.

We acknowledge that this evolutionary interpretation remains speculative, but it provides a conceptual framework consistent with our experimental observations and comparative methylation patterns across animal lineages. We have now clarified this interpretation in the discussion section and emphasized that this model reflects stage-specific de novo methylation activity rather than expression alone determining methylation recovery.

"*Notably, DNMT3 is expressed both early and late in development (Fig. 1c), yet later developmental expression does not appear sufficient to drive substantial 5mC gain in somatic tissues, even after complete regeneration cycles. This may reflect developmental regulation of DNMT3 activity, for example through restricted nuclear localization or reduced enzymatic activity in somatic cells. Possibly, limiting de novo methylation in somatic tissues may have limited evolutionary consequences, as transposable element mobilization in the soma is unlikely to be inherited, while maintenance by DNMT1 may be sufficient to preserve 5mC at essential, highly expressed genes.*"

Reviewer #2 (Remarks on code availability):

The code is actually not available on the link provided. The link does not work.

Response: This was an oversight, as we didn't make the github repository public, please find the code here: https://github.com/AlexdeMendoza/Nematostella_GbM/.

Guynes, Kero, Luke A. Sarre, Allan M. Carrillo-Baltodano, et al. 2024. "Annelid Methylomes Reveal Ancestral Developmental and Aging-Associated Epigenetic Erosion across Bilateria." *Genome Biology* 25 (1): 204.

Pappalardi, Melissa B., Kathryn Keenan, Mark Cockerill, et al. 2021. "Discovery of a First-in-Class Reversible DNMT1-Selective Inhibitor with Improved Tolerability and Efficacy in Acute Myeloid Leukemia." *Nature Cancer* 2 (10): 1002–1017.

Ross, Samuel E., Javier Vázquez-Marín, Krista R. B. Gert, et al. 2023. "Evolutionary Conservation of Embryonic DNA Methylome Remodelling in Distantly Related Teleost Species." *Nucleic Acids Research* 51 (18): 9658–9671.

Our point-by-point response to the reviewers' remarks:

Reviewer #1 (Remarks to the Author):

The authors have provided a clear and convincing response to my major concern regarding the novelty of their work. Their rebuttal appropriately emphasises that the role of gene body methylation in transposable element repression has not been previously documented in invertebrates, where the prevailing paradigm links gbM primarily to actively transcribed genes. The revised abstract now appropriately frames their finding within this evolutionary and ecological context.

While DNA methylation is the focus here, many animals also employ small-RNA-mediated pathways, particularly PIWI–piRNA systems, as a conserved mechanism for transposable element repression; a brief comment on how gbM-mediated repression relates to or differs from piRNA-mediated control would help situate the findings in the broader landscape of TE regulatory mechanisms.

Based on the authors' responses and the revised statements, I believe this manuscript is now suitable for consideration for publication in Nature Ecology & Evolution.

Response: We thank the reviewer for their positive assessment and for this final suggestion. We agree that contextualizing gbM alongside small-RNA pathways provides a comprehensive view of genome defence for the readers. To address this, we have added a new paragraph to the Discussion section to explicitly contrast the sequence-specific, trans-acting nature of piRNAs with the structural, cis-acting protection provided by gbM.

The added text reads as follows:

"*While small-RNA pathways such as the PIWI-piRNA system provide targeted, sequence-specific surveillance against TE transcripts, gbM appears to serve a complementary yet distinct function. The piRNA system primarily acts in trans to degrade specific TE transcripts and guide localized transcriptional silencing. For example, in Nematostella and other cnidarians, piRNAs are enriched during early development and in adult stem cells/germline[47,48]. In contrast, gene body methylation acts in cis as a chromatin feature that broadly suppresses spurious transcriptional initiation within actively transcribed regions, including intragenic TEs. Thus, whereas small RNA pathways primarily combat active transposon mobilization, gbM appears to safeguard the transcriptional integrity of host genes that harbor embedded repetitive elements. Notably, the coupling between small RNA pathways and DNA methylation deposition is thought to represent a later evolutionary innovation, most clearly established in tetrapods[49], suggesting that gbM-mediated repression and piRNA surveillance may operate largely independently in invertebrates.*"

Reviewer #2 (Remarks to the Author):

As I said in my original review I believe this manuscript to be of extremely high quality. In addition it addresses a broadly interesting question and provided an abundance of new data and understanding (within the limits of what can be expected in a single manuscript).

The authors have addressed all of my comments sufficiently. In addition, I believe the authors have also addressed the comments of the other reviewer. I therefore enthusiastically support the publication of this manuscript.

Reviewer #2 (Remarks on code availability):

I am now able to access the associated code and I have looked through briefly. I have not tested the individual code but in a general sense it all seems to be there.

Response: We sincerely thank the review for their enthusiastic support, their highly constructive feedback throughout the review process, and for taking the time to verify the repository and code availability.
